# Burden of disease variants in participants of the long life family Study

**DOI:** 10.18632/aging.100724

**Published:** 2015-02-05

**Authors:** Meredith Stevenson, Harold Bae, Nicole Schupf, Stacy Andersen, Qunyuan Zhang, Thomas Perls, Paola Sebastiani

**Affiliations:** ^1^ Department of Biostatistics, Boston University School of Public Health, Boston, MA 02118, USA; ^2^ College of Public Health and Human Sciences, Oregon State University, OR 97331, USA; ^3^ Department of Neurology, Columbia University, New York City, NY 10027, USA; ^4^ Section of Geriatrics, Department of Medicine, Boston University School of Medicine and Boston Medical Center, Boston, MA 02118, USA; ^5^ Division of Statistical Genomics, Department of Genetics, Washington University School of Medicine, Campus Box 8506, St. Louis, MO 63108; USA

## Abstract

Case control studies of nonagenarians and centenarians provide evidence that long-lived individuals do not differ in the rate of disease associated variants compared to population controls. These results suggest that an enrichment of novel protective variants, rather than a lack of disease associated variants, determine the genetic predisposition to exceptionally long lives. Using data from the Long Life Family Study (LLFS), we sought to replicate these findings and extend them to include a larger number of disease-specific risk alleles. To accomplish this goal, we built a genetic risk score for each of four age-related disease groups: Alzheimer's disease, cardiovascular disease and stroke, type 2 diabetes, and various cancers and compared the distribution of these scores between older participants of the LLFS, their offspring and their spouses. The analyses showed no significant differences in distribution of the genetic risk scores for cardiovascular disease and stroke, type 2 diabetes, or cancer between the groups, while participants of the LLFS appeared to carry an average 1% fewer risk alleles for Alzheimer's disease compared to spousal controls and, while the difference may not be clinically relevant, it was statistically significant. However, the statistical significance between familial longevity and the Alzheimer's disease genetic risk score was lost when a more stringent linkage disequilibrium threshold was imposed to select independent genetic variants.

## INTRODUCTION

Several studies have demonstrated that exceptional longevity can have a strong familial component. Specific sibships have been described in which the presence of multiple siblings achieving extreme old age was extremely unlikely to have occurred by chance and these siblings must have genetic and/or environmental factors in common facilitating such clustering [1]. Sibship studies reveal high relative risks for surviving to 90+ years for the siblings of centenarians and these relative risks increase with the older and older ages of the proband [2–8], thus suggesting an influential genetic component. Tan and colleagues have noted that the power of a sample for the discovery of genetic variants associated with exceptional longevity increases when the sample includes centenarians versus nonagenarians [9]. Finally, the accuracy of a genetic model composed of multiple genetic markers to differentiate between centenarians and referent cohort subjects increased with the ages of the centenarians, especially those surviving beyond 106+ years, suggesting that the genetic component of exceptional longevity (EL) increases with increasing age beyond 100 years [10, 11]. Additionally, centenarians are not only a human model of exceptional longevity, but they are also a human model of healthy aging as many centenarians, and super-centenarians in particular, compress morbidity and disability towards the ends of their lives [12].

Both the New England Centenarian Study and the Leiden Longevity Study have found that genetic variants associated with age-related diseases were just as prevalent in their centenarian and nonagenarian samples as in general population samples [11, 13]. Similar results were recently shown in a small group of centenarians of Ashkenazi Jewish descent [14]. Therefore it seems likely that what sets these individuals apart from those who do not achieve exceptional longevity is, in part, an increased prevalence of protective genetic variants. The increased prevalence of protective genetic variants has also been suggested by work from the Ashkenazi Jewish Centenarian Study in which numerous subjects were observed to achieve extreme old age despite a history or presence of bad health habits otherwise associated with premature mortality. The authors suggest that protective variants must be present to facilitate survival to extreme old age in these subjects [15]. With access to Long Life Family Study (LLFS) data, we set out to determine if the number of disease-associated genetic variants is different between subjects selected because of familial longevity and spousal controls. By developing disease-specific genetic risk scores and utilizing a variety of modeling techniques, we compare the number of age-related disease risk alleles between those with familial longevity and those without to see if a difference exists.

## RESULTS

Table 1 summarizes the characteristics of 1562 LLFS participants in generation one (G1), and 3102 in generation two (G2). Approximately 55% of subjects in generation one died since enrollment and the median age at death was 95 years for males and 97 years for females as of June, 2014.

**Table 1:** Distribution of Generation 1 and 2 Individuals with genotype data

Generation 1 (N = 1562)			
Relation	Number of Individuals (Alive^1^)	Mean Age^2^	Male/Female
Proband	511 (177)	97.8	264/247
Sibling of Proband	862 (419)	93.3	392/470
Half Sibling of Proband	14 (6)	91.6	5/9
Spouse of Proband (control)	37 (22)	91.4	1/36
Spouse of Sibling of Proband (control)	134 (93)	87.3	37/97
Sibling of Spouse of Proband (control)	2 (1)	91.0	2/0
Sibling of Spouse of Sibling of Proband (control)	2 (1)	92.5	0/2

Generation 2 (*N* = 3102)			
Relation	Number of Individuals (Alive^1^)	Mean Age^2^	Male/Female
Offspring of Proband	731 (699)	68.0	283/448
Offspring of Siblings	1577 (1505)	65.8	690/887
Offspring of Half Siblings	13 (12)	72.8	7/6
Half Sibling of Offspring of Proband	1 (1)	55.0	1/0
Half Sibling of Offspring of Sibling	10 (10)	68.7	7/3
Offspring of Sibling of Spouse of Sibling (control)	1 (1)	62.0	1/0
Spouse of Offspring (control)	769 (722)	66.9	409/360

1 Alive as of June 2014

2 Mean Age at Last Contact as of June 2014

We analyzed the four major age-related disease groups: Alzheimer's disease, cardiovascular disease and stroke, type 2 diabetes, and cancers and, using the selection procedure described in the methods, we created a GRS with 93 SNPs associated with Alzheimer's disease (Table S1), a GRS with 239 SNPs associated with cardio-vascular disease and stroke (Table S2), a GRS with 155 SNPs associated with type 2 diabetes (Table S3), and a GRS with 431 SNPs associated with various cancers (Table S4). Supplement Figures S1 through S4 show the distribution of the rate of risk alleles in generation one (proband, siblings and their spouses), and in generation two (offspring and their spouses) for the four disease groups. Summaries of the analysis based on Poisson mixed effects models are displayed in Table 2. Additional results based on the alternative approaches described in the methods are displayed in the Supplemental Online Material (Supplement Tables S6—S12).

**Table 2:** Association of familial longevity with GRS in four age-related disease groups

Alzheimer's Disease									
	LD threshold of r^2^ > 0.8 (93 SNPs)			LD threshold of r^2^ > 0.2 (83 SNPs)			Published GRS (8 SNPs)		
Data	β^1^	Z-Stat	pval	β^1^	Z-stat	pval	β^1^	Z-Stat	pval
Generation One	−0.01512	−1.50	0.133	−0.00622	−0.60	0.546	−0.02100	−0.67	0.503
Generation Two	−0.00974	−1.90	0.057	−0.00473	−0.89	0.371	−0.01230	−0.76	0.447
Both Generations	−0.01067	−2.320	0.020	−0.00568	−1.20	0.230	−0.01535	−1.07	0.285

CVD and Stroke									
	LD threshold of r^2^ > 0.8 (239 SNPs)			LD threshold of r^2^ > 0.2 (218 SNPs)			Published GRS (20 SNPs)		
Data	β^1^	Z-Stat	pval	β^1^	Z-stat	pval	β^1^	Z-Stat	pval
Generation One	−0.00361	−0.62	0.535	−0.00380	−0.62	0.535	0.02640	1.36	0.174
Generation Two	−0.00022	−0.07	0.944	−0.00134	−0.43	0.667	0.00527	0.53	0.596
Both Generations	−0.00119	−0.45	0.653	−0.00211	−0.76	0.447	0.00809	0.92	0.358

Type 2 Diabetes									
	LD threshold of r^2^ > 0.8 (155 SNPs)			LD threshold of r^2^ > 0.2 (137 SNPs)			Published GRS (14 SNPs)		
Data	β^1^	Z-Stat	pval	β^1^	Z-stat	pval	β^1^	Z-Stat	pval
Generation One	0.00319	0.48	0.631	0.00376	0.53	0.596	0.01409	0.64	0.522
Generation Two	0.00410	1.20	0.230	0.00339	0.93	0.352	0.01397	1.23	0.219
Both Generations	0.00348	1.14	0.254	0.00313	0.96	0.337	0.01510	1.50	0.134

Cancer						
	LD threshold of r^2^ > 0.8 (431 SNPs)			LD threshold of r^2^ > 0.2 (386 SNPs)					
Data	β^1^	Z-Stat	pval	β^1^	Z-stat	pval
Generation One	−0.00074	−0.17	0.865	−0.00161	−0.36	0.719
Generation Two	0.00026	0.12	0.905	−0.00052	−0.23	0.818
Both Generations	−0.00019	−0.10	0.920	−0.00083	−0.40	0.689

1 β Estimate is for the regression coefficient for the familial longevity indicator (0 = control, 1 = proband or relative of proband), in log-scale for the analysis of generation one subjects (*N* = 1562), generation two subjects (*N* = 3102), and aggregated data from both generations. The results of generation one and two are adjusted for sex. The results of aggregated data from both generations are adjusted for sex and generation. Results from 3 types of GRS are presented: GRS with LD threshold of *r*^2^ > 0.8 and *r*^2^ > 0.2 included SNPs with LD of 0.8 or less, and 0.2 or less; published GRS were shown to be significantly associated with disease in the literature.

Table 2 reports the results of the association between familial longevity and the GRS for Alzheimer's disease after adjusting for sex. Familial longevity is significantly associated with the genetic risk score in the analysis with aggregated data from the two generations at the 0.05 level (*p*-value 0.020) but did not reach statistical significance in either generation alone, although the association was borderline significant in the offspring with a *p*-value of 0.057. The results from the aggregated data analysis shows that, on average, LLFS individuals with familial longevity carry 1.06% fewer risk alleles than controls since the ratio between expected number of risk alleles is exp(−0.01067) = 0.989. It is interesting to note that once the data from both generations are combined, not only is the association significant at the 0.05 level, but the significance of the association is stronger in the full data than either generation alone. This indicates that an association between GRS and familial longevity in the older generation may actually be present, but, as our power simulations showed, the relatively small sample size did not provide sufficient power to detect that association (Table 4). However, we should also note that when we impose an LD threshold of *r*^2^ > 0.2, the statistical significance of this association is lost. Table 3 shows the results of the analyses in which (1) both SNPs rs2075650 in TOMM40 and rs769449 (which is in high LD with rs2075650) were removed, (2) *rs7412 in APOE was removed*, or (3) all three of these SNPs were removed from the GRS. The association between familial longevity and the score without *rs2075650 in* TOMM40 and rs769449 is borderline significant (*p*-value 0.051) only in the analysis with aggregated data from both generations and the effect is small: LLFS subjects carry on average 0.88% fewer Alzheimer's disease alleles than controls. Removing *rs7412 in APOE* from the genetic score has less of an impact in the full data analysis with a 1.04% smaller rate of disease alleles carried in those with familial longevity compared to controls and statistical significance was retained (*p*-value 0.025). When all three SNPs were removed from the GRS, the association of the GRS with familial longevity was no longer significant in any of the samples – those with familial longevity have, on average, 0.85% fewer risk alleles than controls (*p*-value 0.067).

**Table 3:** Alzheimer's Results from Poisson Generalized Linear Model without APOE SNPs

	Removed rs2075650 & rs769449			Removed rs7412			Removed rs2075650, rs769449, and rs7412		
Data	β^1^	Z-Stat	pval	β^1^	Z-stat	pval	β^1^	Z-Stat	pval
Generation One	−0.01324	−1.34	0.179	−0.01583	−1.55	0.121	−0.01329	−1.30	0.193
Generation Two	−0.00739	−1.46	0.145	−0.00934	−1.79	0.073	−0.00737	−1.41	0.158
Both Generations	−0.00884	−1.95	0.051	−0.01049	−2.25	0.025	−0.00857	−1.83	0.067

1 β Estimate is the regression coefficient for the familial longevity indicator (0 = control, 1 = proband or relative of proband), in log-scale for the analysis of generation one subjects (*N* = 1562), generation two subjects (*N* = 3102), and aggregated data from both generations. As in Table 2, the results of generation one and two are adjusted for sex, while the results of aggregated data from both generations are adjusted for sex and generation. SNP rs2075650 is in *TOMM40* while SNP rs7412 is in *APOE*.

**Table 4:** Results of Power Simulations

1% Difference (β = −0.0101)				
Data	Alzheimer's Disease	CVD and Stroke	Type 2 Diabetes	Cancers
Generation One	0.38	0.73	0.65	0.93
Generation Two	0.78	1.00	1.00	1.00
Both Generations	0.91	1.00	1.00	1.00

5% Difference (β = −0.0513)				
Data	Alzheimer's Disease	CVD and Stroke	Type 2 Diabetes	Cancers
Generation One	1.00	1.00	1.00	1.00
Generation Two	1.00	1.00	1.00	1.00
Both Generations	1.00	1.00	1.00	1.00

10% Difference (β = −0.1054)				
Data	Alzheimer's Disease	CVD and Stroke	Type 2 Diabetes	Cancers
Generation One	1.00	1.00	1.00	1.00
Generation Two	1.00	1.00	1.00	1.00
Both Generations	1.00	1.00	1.00	1.00

Simulations were performed to assess the power to detect 1, 5, and 10 percent differences in number of risk alleles for those with familial longevity compared to those without. The values in the cells indicate the power to detect each difference for all four GRSs.

The results for the GRS of type 2 diabetes are markedly different. Instead of seeing a decreased number of risk alleles for type 2 diabetes in subjects with familial longevity when compared to controls, we actually see a slightly *increased* number of risk alleles within those with familial longevity, and while these results were not statistically significant (*p*-value > 0.1) in the Poisson mixed effects model, Tables S7 and S8 show that, under the Poisson GLM with GEE using an exchangeable correlation structure and the Linear Mixed Effects Model with the Kinship matrix, this association *is* significant at the 0.05 level. This indicates that the number of risk alleles related to type 2 diabetes is not smaller among subjects with familial longevity compared to those without, and may, in fact, be higher. The results of the analyses for cardiovascular disease and stroke show that LLFS participants with familial longevity have, on average, lower genetic risk scores than control individuals. However, these results did not reach statistical significance (smallest *p*-value 0.36 in the analysis of data from both generations using the published type 2 diabetes GRS).

For the analyses of the cancer GRS, the results are somewhat mixed. From Table 2, we see that it appears there is a decreased rate of risk alleles among those with familial longevity compared to controls in the older generation as well as when both generations are combined. However, when looking at the offspring alone, we see a slightly increased rate of risk alleles among those with familial longevity. In all cases, however, the beta coefficients corresponding to familial longevity are very small, so it is not surprising that we see some coefficients greater than 0 and others smaller. This indicates that there is no statistical difference in the number of risk alleles for cancers comparing those with familial longevity to participants without.

Overall, the results were similar from all secondary analyses – when other statistical models were used for all disease-specific genetic risk scores, when the LD threshold was lowered or analyses were limited to SNPs in published GRSs that were shown to be associated with disease risk, and when the controls within generation one were removed and we substituted younger controls from the New England Centenarian Study (see SOM). The most notable difference was that reducing the number SNPs in the Alzheimer's disease GRS resulted in a loss of statistical significance of the association between the GRS and familial longevity. This indicates that our initial results could have been due to the effect of co-segregated SNPs implying that Alzheimer's disease is not an exception to the rule and the number of risk alleles does not significantly differ between controls and individuals with familial longevity.

## DISCUSSION

Our data show that with the potential exception of Alzheimer's disease, LLFS participants with familial longevity do not appear to have fewer age-related disease risk alleles when compared to controls. The results are robust and similar conclusions were reached with alternative statistical analyses described in the SOM.

Sebastiani et al recently reported that compared to subjects not selected for familial longevity, the older generation LLFS subjects have lower hazards for cancer, cardiovascular disease, severe dementia, diabetes, hypertension, osteoporosis, and stroke [16]. The age at which 20% of the LLFS siblings and probands had one or more age-related diseases was approximately 10 years later than the controls. Thus, as with most centenarians, and particularly those beyond the age of 103 years [12], LLFS members of families that cluster for exceptional longevity not only live longer but also have extended health-spans. Also similar to centenarians [11, 13], this “resistance” to age-related diseases that are responsible for much of the morbidity and mortality in the elderly appears not to be associated with a decreased rate of genetic variants previously found to be associated with these diseases. LLFS participants also exhibit lower cognitive impairment in both generations [17] and we recently showed that even compared to centenarians from the New England Centenarian Study, LLFS participants from generation one have significantly lower prevalence of Alzheimer's disease and significantly older age of onset [16]. The GRS for Alzheimer's disease included 93 SNPs pointing to 50 genes. We cannot determine the relative pathogenic contribution of any single variant contributing to the GRS since the score reflects a collective risk.

The increased risk for AD associated with the ApoE ɛ4 allele is well known [18], and its frequency has previously been noted to be very low in centenarians [19, 20]. Schupf et al [21] showed that, compared to their spouses, LLFS offspring had a 30% lower chance of carrying the G allele of SNP rs2075650 in *TOMM40*, which is in moderate linkage disequilibrium with the *APOE* ɛ4 allele. This SNP is one of the 93 SNPs included in the GRS for Alzheimer's disease (Table S1). The additional analyses in which this SNP, rs769449, and/or rs7412 in *APOE* were removed from the GRS for Alzheimer's disease suggest that additional Alzheimer's disease-associated genetic variants appear to be less frequent in the older generation of LLFS participants while generally, for other age-related diseases, such a relative lack of disease associated variants amongst exceptional survivors does not seem to be the case. For example, in more random samples such as the New England Centenarian Study, similar differences in rates of Alzheimer's and dementia associated alleles were not noted [11]. One of the eligibility requirements of LLFS was that the proband and at least one living sibling were able to provide informed consent and therefore had some cognitive competence at old age [22]. This requirement may have resulted in a sample of exceptional survivors with a lack of Alzheimer's disease predisposing variants.

A limitation of this study is that we cannot exclude with certainty that the spouses of probands, their siblings and offspring in the LLFS have familial longevity. Inclusion of such controls would bias our results toward the null hypothesis of no difference with respect to number of risk alleles and invalidate the conclusions. Probands of the LLFS were enrolled based on evidence of familial longevity that was scored using the Familial Longevity Selection Score (FLoSS) [23], and families with an eligible score are very rare in the population. For example, we estimated that fewer than one percent of families enrolled in the Framingham Heart Study would achieve a FLoSS that makes them eligible to be in the LLFS. Therefore, the likelihood that the spousal controls have longevity running in their own families to the degree observed in LLFS families is unlikely.

The results of our power simulations (Table 4) indicate that, given our sample size, we had sufficient power to detect a 1% difference in GRSs comparing those with familial longevity to controls – had it been present. Thus, the results generated from our analyses of the genetic risk scores related to cardiovascular disease and stroke, type 2 diabetes, and cancer support the hypothesis that individuals with familial longevity do not have a smaller number of age-related disease risk alleles compared to controls. Therefore it is likely, as has been posited by the New England Centenarian Study [10, 11] and Leiden Longevity Study [13], that people with familial longevity have a relatively increased prevalence of protective genetic and environmental factors that confer decreased risk for the diseases that we looked at in this study. Uncovering these protective factors could lead to screening, prevention strategies and perhaps even therapeutic interventions to facilitate healthy aging. Another point to stress is the predictive value of disease-associated variants or lack thereof in assessing a person's risk of developing age-related diseases. Our findings suggest that single variants, particularly in isolation and not interpreted in the context of other disease associated variants or protective variants are highly unreliable predictors.

Finally, understanding the epidemiologic relative importance of disease associated and protective variants may lend clues to drugable pathways and targets.

## METHODS

### Subjects and Genotype Data

The LLFS enrolled 583 families with evidence of familial longevity based on the Family Longevity Selection Score [23]. Family members included probands, siblings and their spouses (generation G1), their offspring and spouses (generation G2). Enrollment occurred between 2006 and 2009 and participants have been followed annually since 2010. Table 1 provides a summary of the participants included in the analysis. DNA samples of participants were genotyped at the Center for Inherited Disease Research (CIDR) using the Illumina Omni 2.5 platform, and genotype calls were cleaned following a strict quality control process described in [24]. Genotype data were imputed to the 1000 genomes using MACH (http://www.sph.umich.edu/csg/abecasis/MACH/download/). Genotyped data are available from dbGaP (phs000397.v1.p1).

### Calculation of Disease-Specific Genetic Risk Scores

The list of single nucleotide polymorphisms (SNPs) associated with specific age-related diseases was based on the Catalog of Published Genome-Wide Association Studies (http://www.genome.gov/26525384) compiled by The National Human Genome Research Institute and was downloaded on July 26, 2014 [25]. From this catalog, a list of 1143 SNPs that were genome-wide significantly associated (*p* < 5 x10^−8^) with age-related diseases and had reported allele(s) associated with disease was compiled. Four disease-specific genetic risk scores (GRSs) were created for the main age-associated diseases: Alzheimer's disease (Table S1), cardio-vascular diseases and stroke (Table S2), type 2 diabetes (Table S3), and cancers (Table S4). SNPs that were listed as having more than two common variants using the SNPper annotation tool (http://SNPper.chip.org) were omitted. To avoid inflating the effects of some loci that were overrepresented by many SNPs in strong linkage disequilibrium (LD), one of each pair of SNPs that were in LD (*r*^2^ > 0.8) were randomly removed. A total of 20 SNPs associated with Alzheimer's disease, 58 associated with all types of cancer, 90 associated with cardiovascular disease and 55 associated with type 2 diabetes were removed with this filtering. In the case of Alzheimer's disease, the Catalog of Published Genome-Wide Association Studies contained 103 associated SNPs. Additional literature review revealed 10 SNPs associated with Alzheimer's disease that had not been included in the Catalog and these SNPs were also included in our list [26–32]. This procedure identified a total of 93 SNPs associated with Alzheimer's disease, 239 SNPs associated with cardiovascular disease and stroke, 155 SNPs associated with type 2 diabetes, and 431 SNPs associated with various cancers for a total of *N* = 918 SNPs. Genetic risk scores for each LLFS participant *i* were computed as:

$$GRS_{i}=\sum_{j=1}^{m}x_{ij}$$

where *x*_ij_ is the number of risk alleles (0, 1, 2) at the *j*^th^ SNP, and *m* is the total number of SNPs within each disease group (e.g. *m* = 93 for the Alzheimer's disease GRS). We computed unweighted scores to focus attention on the absolute number of disease alleles rather than their estimated genetic effect.

### Statistical Analysis

The distributions of GRSs for each age-related disease were compared between members of families selected for longevity and spouses using Poisson regression with a log-linear link. The four genetic risk scores were the outcome variable in all the analyses and, because the GRS is an integer, we assumed that this variable followed a Poisson distribution. To avoid loss of power due to missing values, we used an offset term equal to the log of twice the number of SNPs in the GRS for which each person had genetic data. Thus, the rate of risk alleles was modeled as opposed to the count of risk alleles. We created a binary variable to indicate whether or not a person was a proband or genetically related to a proband (1 = yes, 0 = no). Those who married into the proband's family were considered controls in all of the analyses (See Table 1). This indicator variable represented familial longevity in the regression analyses, and the exponential of the regression coefficient represents the ratio of risk alleles between subjects with and without familial longevity. We first performed generation-specific analyses and then performed full data analyses adjusting by generation. In each analysis, we estimated the crude relationship between familial longevity and GRS and then adjusted the association for sex. Age at enrollment was not included because it is correlated with the indicator of familial longevity. To account for the correlation among individuals, we fit Poisson linear mixed effects models with a log-link function, an offset equal to the number of SNPs included in each person's GRS, and a random intercept for each family using the g*lmer* function in *lme4* package of the R statistical software. In these analyses, R automatically determined the correlation structure based on the random effects. All analyses were performed in R version 3.0.2. Statistical significance of the coefficients was tested using the likelihood ratio test [33]. To assess the robustness of the results to the model specification, we also conducted additional analyses using (1) Generalized Linear Models in which the within-family correlation was ignored; (2) Generalized Estimating Equations with a sandwich estimator to reduce the inflation of the test statistics compared to standard Generalized Linear Models, and (3) Linear Mixed Effects Models with kinship correction assuming an approximate normal distributions for the rate of risk alleles. The results of these three additional analyses are in the supplementary material (Supplement Tables 6, 7, 8) and were similar to the analysis based on Poisson linear mixed effects model analysis. In addition to the previously described methods, a thorough sensitivity analysis was conducted.

To confirm that our results were not a product of our rather lenient LD threshold, we ran a secondary analysis where one SNP from each pair of SNPs with an *r*^2^ > 0.2 was randomly deleted. This additional filtering left us with 83 SNPs associated with Alzheimer's disease, 218 SNPs in our GRS for cardiovascular disease and stroke, 137 SNPs associated with type 2 diabetes, and 386 SNPs associated with various cancers. Results of these analyses are displayed in Table 2 as well as Tables S6-S12.

We also performed a literature review to identify published genetic risk scores that had been shown to accurately discriminate cases and non-cases for three of our four age-related diseases: Alzheimer's disease [34], cardiovascular disease [35], and type 2 diabetes [36]. Because our GRS for cancer contains various types of cancer, we were unable to find a published genetic risk score of the same scope for discriminating between cases and controls. We then re-ran analyses limiting to SNPs in each of the published GRSs. In Verhaaren et al.'s paper [34], 11 SNPs were included in their GRS that was associated with prevalent Alzheimer's disease. Of these 11 SNPs, two of the SNPs were removed from our analyses because the GWAS catalog did not list a risk allele and a third was removed due to ambiguous coding, leaving us with 8 SNPs in our GRS for Alzheimer's disease. 29 SNPs were used by Thanassoulis et al. to construct a GRS that was associat-ed with prevalent cardiovascular disease [35]. From these 29 SNPs, we excluded 6 SNPs from our analyses due to multiple risk alleles recorded in the GWAS catalog. An additional 3 SNPs were removed due to poor imputation quality or ambiguous coding. Thanassoulis et al. also saw statistically significant associations between a 13 SNP GRS and prevalent cardiovascular disease, of these 13 SNPs, 12 were included in our secondary analyses. A genetic risk score comprised of 18 SNPs was published by Meigs et al. after being shown to be associated with prevalent type 2 diabetes [36]. Of these 18 SNPs, our GRS contained 14 – one was excluded because no risk allele was supplied in the GWAS catalog and another three were ambiguously coded and were thus removed. Results from these analyses were consistent and are shown in Tables 2 and S6-S12.

Finally, it was of concern that the controls for generation one were nearly as old as the participants with familial longevity which may have accounted for their relatively similar distribution of age-related disease risk alleles. Thus, as another test of the robustness of our results, all previously described methods were repeated in a dataset where the controls for generation one were replaced with the control participants from the New England Centenarian Study (NECS) [10]. The NECS controls were, on average, 8.6 years younger than the LLFS generation one controls. The GRSs in these analyses were limited to those SNPs commonly available between the LLFS data and NECS data (numbers shown in supplementary tables). The corresponding results were concordant with those using only LLFS participants (see Tables S9-S12).

To ensure that we had sufficient power to detect a difference in GRSs between individuals with familial longevity and controls, should it exist, we ran power simulations. For these power simulations, we used the family structure and sample size of the LLFS. We then generated a random intercept for each family and built a linear predictor for each individual using this random intercept and beta coefficients for the effect of familial longevity corresponding to 1, 5, and 10 percent differences between GRSs. For example, for a 1% difference in GRS comparing those with familial longevity to those without, the beta coefficient was log(0.99) = −0.0101. Then, for each individual, their simulated GRS was drawn from a Poisson distribution with λ equal to the exponential of their linear predictor. We then fit a Poisson mixed effects model with a random intercept per family and assessed whether or not the familial longevity indicator was significantly associated with the GRS. This process was repeated 100 times for each GRS. The results of these simulations are shown in Table 4.

## SUPPLEMENTARY TABLES AND FIGURES


