# Erratum: Burden of disease variants in participants of the long life family study

**DOI:** 10.18632/aging.100761

**Published:** 2015-02-05

**Authors:** Meredith Stevenson, Harold Bae, Nicole Schupf, Stacy Andersen, Qunyuan Zhang, Thomas Perls, Paola Sebastiani

Erratum: Burden of disease variants in participants of the long life family study

Meredith Stevenson, Harold Bae, Nicole Schupf, Stacy Andersen, Qunyuan Zhang, Thomas Perls, and Paola Sebastiani

Aging (Albany NY) 2015; 7(2):123-132.

PMCID: PMC 4359694 PMID: 25664523

Information of **Funding** section is not accurate. The correct information on Grant is provided below:

