# Supplementary material for: Burden of disease variants in participants of the long life family Study
Source: Aging (Albany NY). 2015 Feb 5;7(2):123–32. doi: 10.18632/aging.100724 (PMC4359694; doi:10.18632/aging.100724)

**Burden of Disease Variants in Participants of the Long Life Family Study**

**Supplemental Online Material**

Note that in Tables S1-S3 the following scheme was used to denote each Genetic Risk Score (GRS)

- GRS1: LLFS data, LD threshold of r^2^ > 0.8
- GRS2: LLFS data, LD threshold of r^2^ > 0.2
- GRS3: LLFS data, Published GRS (note that this is absent in Table S4 for Cancer)
- GRS4: LLFS & NECS data, LD threshold of r^2^ > 0.8
- GRS5: LLFS & NECS data, LD threshold of r^2^ > 0.2
- GRS6: LLFS & NECS data, Published GRS (note that this is absent in Table S4 for Cancer)

**Table S1**

| **SNP** | **Risk Allele** | **Gene** | **Band** | **Alleles** | **In GRS1** | **In GRS2** | **In GRS3** | **In GRS4** | **In GRS5** | **In GRS6** |
| --- | --- | --- | --- | --- | --- | --- | --- | --- | --- | --- |
| rs10219670 | C |  | 12q23.3 | A/C | 1 | 1 | 0 | 1 | 1 | 0 |
| rs10273775 | G | CNTNAP2 | 7q35 | A/G | 1 | 1 | 0 | 1 | 1 | 0 |
| rs1031261 | C | TTC27 | 2p22.3 | C/G | 0 | 0 | 0 | 0 | 0 | 0 |
| rs10498633 | G | SLC24A4 | 14q32.12 | G/T | 1 | 1 | 0 | 1 | 1 | 0 |
| rs10751667 | T | AP2A2 | 11p15.5 | A/T | 0 | 0 | 0 | 0 | 0 | 0 |
| rs10792832 | G |  | 11q14.2 | A/G | 0 | 0 | 0 | 0 | 0 | 0 |
| rs10838725 | C | CELF1 | 11p11.2 | C/T | 1 | 1 | 0 | 1 | 1 | 0 |
| rs10948363 | G | CD2AP | 6p12.3 | A/G | 0 | 0 | 0 | 0 | 0 | 0 |
| rs11136000 | C | CLU | 8p21.1 | C/T | 1 | 1 | 1 | 1 | 1 | 1 |
| rs11139399 | C |  | 9q21.32 | C/T | 1 | 1 | 0 | 1 | 1 | 0 |
| rs11218343 | T | SORL1 | 11q24.1 | C/T | 1 | 1 | 0 | 1 | 1 | 0 |
| rs113524839 | T |  |  |  | 1 | 1 | 0 | 1 | 1 | 0 |
| rs115550680 | G |  |  |  | 0 | 0 | 0 | 0 | 0 | 0 |
| rs11754661 | A | MTHFD1L | 6q25.1 | A/G | 1 | 1 | 0 | 1 | 1 | 0 |
| rs11771145 | G | EPHA1 | 7q35 | A/G | 1 | 1 | 0 | 1 | 1 | 0 |
| rs11848070 | C | PCNX | 14q24.2 | A/G | 1 | 1 | 0 | 0 | 0 | 0 |
| rs11889338 | A |  | 2p24.2 | A/G | 1 | 1 | 0 | 1 | 1 | 0 |
| rs11983798 | T | ATXN7L1 | 7q22.3 | A/G | 1 | 1 | 0 | 0 | 0 | 0 |
| rs12361953 | C | LUZP2 | 11p14.3 | C/G | 0 | 0 | 0 | 0 | 0 | 0 |
| rs13037749 | A | CHD6 | 20q12 | A/G | 1 | 1 | 0 | 1 | 1 | 0 |
| rs1422438 | G |  | 5q21.3 | A/C | 1 | 1 | 0 | 1 | 1 | 0 |
| rs145848414 | A |  |  |  | 0 | 0 | 0 | 0 | 0 | 0 |
| rs1476679 | T | ZCWPW1 | 7q22.1 | C/T | 1 | 1 | 0 | 1 | 1 | 0 |
| rs17006206 | G | SLC4A1AP | 2p23.2 | A/G | 1 | 1 | 0 | 0 | 0 | 0 |
| rs17125944 | C | FERMT2 | 14q22.1 | C/T | 1 | 1 | 0 | 1 | 1 | 0 |
| rs17178006 | G | MSRB3 | 12q14.3 | G/T | 1 | 1 | 0 | 0 | 0 | 0 |
| rs17189298 | A |  | 2q14.2 | A/G | 1 | 1 | 0 | 1 | 1 | 0 |
| rs17511627 | C |  | 13q12.13 | A/C | 1 | 1 | 0 | 1 | 1 | 0 |
| rs17767225 | T |  | 14q24.2 | C/T | 1 | 0 | 0 | 0 | 0 | 0 |
| rs190982 | A |  | 5q14.3 | A/G | 1 | 1 | 0 | 1 | 1 | 0 |
| rs1923775 | T | POLN | 4p16.3 | C/T | 1 | 1 | 0 | 1 | 1 | 0 |
| rs1997111 | T |  | 12q24.23 | G/T | 1 | 1 | 0 | 1 | 1 | 0 |
| rs2075650 | G | TOMM40 | 19q13.32 | A/G | 1 | 1 | 0 | 1 | 1 | 0 |
| rs2298948 | C | C2orf3 | 2p12 | A/G | 1 | 1 | 0 | 1 | 1 | 0 |
| rs2337406 | C |  | 14q32.33 | A/G | 0 | 0 | 0 | 0 | 0 | 0 |
| rs2373115 | G | GAB2 | 11q14.1 | G/T | 1 | 1 | 0 | 1 | 1 | 0 |
| rs24449894 | A |  |  |  | 0 | 0 | 0 | 0 | 0 | 0 |
| rs2718058 | A |  | 7p14.1 | A/G | 1 | 1 | 0 | 1 | 1 | 0 |
| rs2838923 | A | NCRNA00175 | 21q22.3 | A/G | 1 | 1 | 0 | 1 | 1 | 0 |
| rs28834970 | C | PTK2B | 8p21.2 | C/T | 1 | 1 | 0 | 1 | 1 | 0 |
| rs2935776 | C |  | 8q23.1 | C/T | 1 | 1 | 0 | 1 | 1 | 0 |
| rs35349669 | T | INPP5D | 2q37.1 | C/T | 1 | 1 | 0 | 1 | 1 | 0 |
| rs3752246 | G | ABCA7 | 19p13.3 | C/G | 0 | 0 | 0 | 0 | 0 | 0 |
| rs3764650 | G | ABCA7 | 19p13.3 | G/T | 1 | 1 | 1 | 1 | 1 | 1 |
| rs3820201 | A | SLC1A7 | 1p32.3 | A/G | 1 | 1 | 0 | 1 | 1 | 0 |
| rs3851179 | G |  | 11q14.2 | A/G | 1 | 1 | 1 | 1 | 1 | 1 |
| rs3865444 | C | CD33 | 19q13.41 | G/T | 1 | 1 | 1 | 1 | 1 | 1 |
| rs4145462 | T | MPZL1 | 1q24.2 | A/G | 1 | 1 | 0 | 1 | 1 | 0 |
| rs41458646 | G |  | 2p24.1 | A/G | 1 | 1 | 0 | 1 | 1 | 0 |
| rs4147929 | A | ABCA7 | 19p13.3 | A/G | 1 | 0 | 0 | 1 | 0 | 0 |
| rs429358 | C | APOE | 19q13.32 | C/T | 1 | 0 | 0 | 1 | 0 | 0 |
| rs4298437 | A | RELN | 7q22.1 | C/T | 1 | 1 | 0 | 1 | 1 | 0 |
| rs4667682 | C |  | 2q31.1 | C/T | 1 | 1 | 0 | 1 | 1 | 0 |
| rs4676049 | A |  | 2q12.3 | C/T | 1 | 1 | 0 | 1 | 1 | 0 |
| rs472926 | C | CDON | 11q24.2 | A/G | 1 | 1 | 0 | 0 | 0 | 0 |
| rs4925189 | G | CDH4 | 20q13.33 | A/G | 1 | 1 | 0 | 1 | 1 | 0 |
| rs4937314 | C |  | 11q24.3 | A/G | 1 | 1 | 0 | 0 | 0 | 0 |
| rs509208 | G |  | 3q26.1 | C/G | 0 | 0 | 0 | 0 | 0 | 0 |
| rs514716 | G | GLIS3 | 9p24.2 | A/G | 1 | 1 | 0 | 1 | 1 | 0 |
| rs519113 | G | PVRL2 | 19q13.32 | C/G | 0 | 0 | 0 | 0 | 0 | 0 |
| rs56131196 | A | APOC1 | 19q13.32 | A/G | 1 | 0 | 0 | 0 | 0 | 0 |
| rs59007384 | T | TOMM40 | 19q13.32 | G/T | 1 | 0 | 0 | 1 | 0 | 0 |
| rs5998432 | T | RFPL3 | 22q12.3 | C/T | 1 | 1 | 0 | 1 | 1 | 0 |
| rs6085820 | A |  | 20p12.3 | G/T | 1 | 1 | 0 | 1 | 1 | 0 |
| rs610932 | G | MS4A6A | 11q12.2 | A/C | 1 | 1 | 1 | 1 | 1 | 1 |
| rs62209 | C | LOC254312 | 10p14 | G/T | 1 | 1 | 0 | 1 | 1 | 0 |
| rs6448799 | T |  | 4p15.33 | C/T | 1 | 1 | 0 | 1 | 1 | 0 |
| rs6468852 | G |  | 8q22.3 | A/G | 1 | 1 | 0 | 1 | 1 | 0 |
| rs6581612 | C |  | 12q14.3 | A/C | 1 | 0 | 0 | 1 | 0 | 0 |
| rs6656401 | A | CR1 | 1q32.2 | A/G | 1 | 1 | 0 | 1 | 1 | 0 |
| rs6678275 | C |  | 1q31.2 | C/G | 0 | 0 | 0 | 0 | 0 | 0 |
| rs668387 | C | SORL1 | 11q24.1 | A/G | 1 | 1 | 0 | 1 | 1 | 0 |
| rs6701713 | A | CR1 | 1q32.2 | A/G | 0 | 0 | 0 | 0 | 0 | 0 |
| rs6703865 | A | SELP | 1q24.2 | A/G | 1 | 1 | 0 | 1 | 1 | 0 |
| rs6733839 | T |  | 2q14.3 | C/T | 1 | 0 | 0 | 1 | 0 | 0 |
| rs6738181 | A |  | 2q33.3 | A/G | 1 | 1 | 0 | 1 | 1 | 0 |
| rs6741949 | G | DPP4 | 2q24.2 | C/G | 0 | 0 | 0 | 0 | 0 | 0 |
| rs6857 | T | TOMM40 | 19q13.32 | A/G | 1 | 1 | 0 | 1 | 1 | 0 |
| rs6859 | A | PVRL2 | 19q13.32 | A/G | 1 | 1 | 0 | 1 | 1 | 0 |
| rs6922617 | A |  | 6p21.1 | A/G | 1 | 1 | 0 | 1 | 1 | 0 |
| rs7039300 | G |  | 9p23 | G/T | 1 | 1 | 0 | 1 | 1 | 0 |
| rs7225151 | A | C17orf87 | 17p13.2 | A/G | 1 | 1 | 0 | 1 | 1 | 0 |
| rs727153 | C |  | 4q32.1 | A/G | 1 | 1 | 0 | 1 | 1 | 0 |
| rs7274581 | T | CASS4 | 20q13.31 | C/T | 1 | 1 | 0 | 1 | 1 | 0 |
| rs72807343 | T | MGAT4B | 5q35.3 | C/T | 1 | 1 | 0 | 1 | 1 | 0 |
| rs7294919 | T | HRK | 12q24.22 | C/T | 1 | 1 | 0 | 0 | 0 | 0 |
| rs7295246 | G |  | 12q12 | G/T | 1 | 1 | 0 | 1 | 1 | 0 |
| rs7412 | C | APOE | 19q13.32 | C/T | 1 | 1 | 0 | 1 | 1 | 0 |
| rs744373 | C |  | 2q14.3 | C/T | 1 | 1 | 1 | 1 | 1 | 1 |
| rs74615166 | C | TRIP4 | 15q22.31 | C/T | 1 | 1 | 0 | 0 | 0 | 0 |
| rs7561528 | A |  | 2q14.3 | A/G | 1 | 0 | 0 | 1 | 0 | 0 |
| rs75932628 | T | TREM2 | 6p21.1 | C/T | 1 | 1 | 0 | 0 | 0 | 0 |
| rs7638995 | A | LMOD3 | 3p14.1 | A/C | 1 | 1 | 0 | 1 | 1 | 0 |
| rs769449 | A | APOE | 19q13.32 | A/G | 1 | 0 | 0 | 1 | 0 | 0 |
| rs7702276 | T |  | 5q11.2 | C/T | 1 | 1 | 0 | 1 | 1 | 0 |
| rs7818382 | T | C8orf38 | 8q22.1 | C/T | 1 | 1 | 0 | 1 | 1 | 0 |
| rs7852872 | C | ASTN2 | 9q33.1 | C/G | 0 | 0 | 0 | 0 | 0 | 0 |
| rs7920721 | G |  | 10p14 | A/G | 1 | 1 | 0 | 1 | 1 | 0 |
| rs8035452 | T | SPPL2A | 15q21.2 | C/T | 1 | 1 | 0 | 1 | 1 | 0 |
| rs9271192 | C |  |  |  | 0 | 0 | 0 | 0 | 0 | 0 |
| rs9315702 | A | LHFP | 13q14.11 | A/C | 1 | 1 | 0 | 1 | 1 | 0 |
| rs9331896 | T | CLU | 8p21.1 | A/G | 0 | 0 | 0 | 0 | 0 | 0 |
| rs9349407 | C | CD2AP | 6p12.3 | C/G | 0 | 0 | 0 | 0 | 0 | 0 |
| rs9381040 | C | TREML2 | 6p21.1 | C/T | 1 | 1 | 0 | 1 | 1 | 0 |
| rs9384488 | A |  | 6q25.3 | A/G | 1 | 1 | 0 | 1 | 1 | 0 |
| rs956225 | A |  | 8q24.13 | A/G | 1 | 1 | 0 | 1 | 1 | 0 |
| rs959695 | C | VPS13B | 8q22.2 | A/G | 1 | 1 | 0 | 1 | 1 | 0 |
| rs983392 | A |  | 11q12.2 | A/G | 1 | 0 | 0 | 1 | 0 | 0 |
| rs9877502 | A |  | 3q28 | A/G | 1 | 1 | 0 | 0 | 0 | 0 |
| rs9969729 | A |  | 9q31.2 | A/G | 1 | 1 | 0 | 1 | 1 | 0 |
| rs597668 | C | EXOC3L2 | 19q13.32 | C/T | 1 | 1 | 1 | 1 | 1 | 1 |
| rs670139 | T |  | 11q12.2 | A/C | 1 | 1 | 1 | 1 | 1 | 1 |

**Table S2**

| **SNP** | **Risk Allele** | **Gene** | **Band** | **Alleles** | **In GRS1** | **In GRS2** | **In GRS3** | **In GRS4** | **In GRS5** | **In GRS6** |
| --- | --- | --- | --- | --- | --- | --- | --- | --- | --- | --- |
| rs10021303 | A | BMPR1B | 4q22.3 | A/G | 1 | 1 | 0 | 1 | 1 | 0 |
| rs10026364 | T |  | 4q28.3 | C/T | 1 | 1 | 0 | 0 | 0 | 0 |
| rs10033464 | T |  | 4q25 | G/T | 1 | 0 | 0 | 0 | 0 | 0 |
| rs1005224 | T | TTLL5 | 14q24.3 | A/T | 0 | 0 | 0 | 0 | 0 | 0 |
| rs10057565 | T | CDH9 | 5p14.1 | C/T | 1 | 1 | 0 | 1 | 1 | 0 |
| rs10091374 | A |  | 8q13.3 | A/T | 0 | 0 | 0 | 0 | 0 | 0 |
| rs10179686 | T | LOC151162 | 2q21.3 | C/T | 1 | 1 | 0 | 1 | 1 | 0 |
| rs10199768 | T | APOB | 2p24.1 | G/T | 1 | 1 | 0 | 1 | 1 | 0 |
| rs10266254 | A | DPY19L2P4 | 7q21.13 | A/G | 0 | 0 | 0 | 0 | 0 | 0 |
| rs1028771 | A | LAMC2 | 1q25.3 | A/C | 0 | 0 | 0 | 0 | 0 | 0 |
| rs10428132 | T | SCN10A | 3p22.2 | G/T | 1 | 1 | 0 | 1 | 1 | 0 |
| rs104623359 | A |  |  |  | 0 | 0 | 0 | 0 | 0 | 0 |
| rs10498345 | T |  | 14q21.1 | A/T | 0 | 0 | 0 | 0 | 0 | 0 |
| rs10502575 | A | MCART2 | 18q12.1 | A/G | 1 | 1 | 0 | 1 | 1 | 0 |
| rs10757274 | G | CDKN2BAS | 9p21.3 | A/G | 0 | 0 | 0 | 0 | 0 | 0 |
| rs10757278 | G | CDKN2BAS | 9p21.3 | A/G | 0 | 0 | 0 | 0 | 0 | 0 |
| rs10790162 | A | BUD13 | 11q23.3 | A/G | 0 | 0 | 0 | 0 | 0 | 0 |
| rs10803016 | T | PLD5 | 1q43 | G/T | 1 | 1 | 0 | 1 | 1 | 0 |
| rs10821415 | A | C9orf3 | 9q22.32 | A/C | 1 | 1 | 0 | 1 | 1 | 0 |
| rs10824026 | A | SYNPO2L | 10q22.2 | A/G | 1 | 1 | 0 | 1 | 1 | 0 |
| rs10830956 | C |  | 11q14.3 | C/T | 0 | 0 | 0 | 0 | 0 | 0 |
| rs10830962 | G | MTNR1B | 11q14.3 | C/G | 0 | 0 | 0 | 0 | 0 | 0 |
| rs10838681 | G | NR1H3 | 11p11.2 | A/G | 1 | 1 | 0 | 1 | 1 | 0 |
| rs10861032 | C |  | 12q23.3 | C/T | 1 | 1 | 0 | 1 | 1 | 0 |
| rs10895547 | T | PDGFD | 11q22.3 | C/T | 1 | 1 | 0 | 1 | 1 | 0 |
| rs10911021 | C |  | 1q25.3 | C/T | 1 | 1 | 0 | 1 | 1 | 0 |
| rs10933436 | A | INPP5D | 2q37.1 | A/C | 1 | 1 | 0 | 1 | 1 | 0 |
| rs10953541 | C | BCAP29 | 7q22.3 | C/T | 1 | 1 | 1 | 1 | 1 | 1 |
| rs11014166 | A | CACNB2 | 10p12.31 | A/T | 0 | 0 | 0 | 0 | 0 | 0 |
| rs11046430 | T |  | 12p12.1 | C/T | 1 | 1 | 0 | 1 | 1 | 0 |
| rs11054731 | A | LRP6 | 12p13.2 | A/G | 1 | 1 | 0 | 1 | 1 | 0 |
| rs11066015 | A | ACAD10 | 12q24.12 | A/G | 0 | 0 | 0 | 0 | 0 | 0 |
| rs11066280 | A | C12orf51 | 12q24.13 | A/T | 0 | 0 | 0 | 0 | 0 | 0 |
| rs11079321 | A | MSI2 | 17q22 | A/G | 1 | 1 | 0 | 1 | 1 | 0 |
| rs1110183 | G |  | 9p13.1 | A/G | 1 | 1 | 0 | 1 | 1 | 0 |
| rs11206510 | T | PCSK9 | 1p32.3 | C/T | 1 | 1 | 1 | 0 | 0 | 0 |
| rs1152591 | A | SYNE2 | 14q23.2 | C/T | 1 | 1 | 0 | 1 | 1 | 0 |
| rs11646213 | T |  | 16q23.3 | A/T | 0 | 0 | 0 | 0 | 0 | 0 |
| rs11651708 | A | PRKCA | 17q24.2 | A/T | 0 | 0 | 0 | 0 | 0 | 0 |
| rs11669133 | A | SMARCA4 | 19p13.2 | A/G | 1 | 1 | 0 | 1 | 1 | 0 |
| rs11671653 | A | MIR638 | 19p13.2 | A/G | 1 | 1 | 0 | 1 | 1 | 0 |
| rs11684202 | A | DTNB | 2p23.3 | A/G | 1 | 1 | 0 | 1 | 1 | 0 |
| rs11708996 | C | SCN5A | 3p22.2 | C/G | 0 | 0 | 0 | 0 | 0 | 0 |
| rs1173771 | G |  | 5p13.3 | C/T | 1 | 1 | 0 | 1 | 1 | 0 |
| rs11752643 | T |  |  |  | 0 | 0 | 0 | 0 | 0 | 0 |
| rs11754288 | A | SLC17A4 | 6p22.2 | A/G | 1 | 1 | 0 | 1 | 1 | 0 |
| rs11760067 | T |  | 6q23.2 | C/T | 1 | 1 | 0 | 1 | 1 | 0 |
| rs11775334 | A | MSRA | 8p23.1 | A/G | 1 | 1 | 0 | 1 | 1 | 0 |
| rs11777747 | T | FLJ43860 | 8q24.3 | C/T | 1 | 1 | 0 | 1 | 1 | 0 |
| rs11781551 | A |  | 8q24.13 | A/G | 1 | 1 | 0 | 1 | 1 | 0 |
| rs11820589 | A | BUD13 | 11q23.3 | A/G | 1 | 0 | 0 | 1 | 0 | 0 |
| rs11825259 | A |  | 11p13 | A/G | 1 | 1 | 0 | 0 | 0 | 0 |
| rs11886999 | T | STARD7 | 2q11.2 | C/T | 1 | 1 | 0 | 1 | 1 | 0 |
| rs1190739 | T |  | Xq26.3 | C/T | 0 | 0 | 0 | 0 | 0 | 0 |
| rs11920719 | A | TNIK | 3q26.2 | A/C | 0 | 0 | 0 | 0 | 0 | 0 |
| rs11921014 | A |  | 3q23 | A/C | 1 | 1 | 0 | 0 | 0 | 0 |
| rs11953630 | T |  | 5q33.3 | C/T | 1 | 1 | 0 | 1 | 1 | 0 |
| rs11984041 | A | HDAC9 | 7p21.1 | C/T | 1 | 1 | 0 | 1 | 1 | 0 |
| rs1198872 | T | ATP6V1C2 | 2p25.1 | C/T | 1 | 1 | 0 | 1 | 1 | 0 |
| rs12098973 | G | NTM | 11q25 | A/G | 1 | 1 | 0 | 1 | 1 | 0 |
| rs12205331 | T | ANKS1A | 6p21.31 | C/T | 0 | 0 | 0 | 0 | 0 | 0 |
| rs12239436 | A |  | 1p32.3 | A/G | 1 | 1 | 0 | 0 | 0 | 0 |
| rs12269901 | C | SIK3 | 11q23.3 | C/G | 0 | 0 | 0 | 0 | 0 | 0 |
| rs12280753 | T | BUD13 | 11q23.3 | C/T | 1 | 1 | 0 | 1 | 1 | 0 |
| rs12286037 | C | ZNF259 | 11q23.3 | C/T | 1 | 0 | 0 | 1 | 0 | 0 |
| rs1231206 | A | SMG6 | 17p13.3 | C/T | 0 | 0 | 0 | 0 | 0 | 0 |
| rs12318506 | T | CAPS2 | 12q21.2 | G/T | 1 | 1 | 0 | 1 | 1 | 0 |
| rs12425791 | A |  | 12p13.33 | A/G | 1 | 1 | 0 | 0 | 0 | 0 |
| rs12524865 | C |  | 6q23.2 | A/C | 1 | 1 | 0 | 1 | 1 | 0 |
| rs12526453 | C | PHACTR1 | 6p24.1 | C/G | 0 | 0 | 0 | 0 | 0 | 0 |
| rs12541335 | G | PHYHIP | 8p21.3 | A/G | 1 | 1 | 0 | 1 | 1 | 0 |
| rs12564445 | A | TNNT2 | 1q32.1 | A/G | 1 | 1 | 0 | 1 | 1 | 0 |
| rs12588287 | T | ATXN3 | 14q32.12 | C/T | 1 | 1 | 0 | 1 | 1 | 0 |
| rs12595292 | A | PLEKHO2 | 15q22.31 | A/G | 1 | 1 | 0 | 1 | 1 | 0 |
| rs1260326 | T | GCKR | 2p23.3 | C/T | 1 | 1 | 0 | 1 | 1 | 0 |
| rs1263173 | A |  | 11q23.3 | A/G | 1 | 0 | 0 | 0 | 0 | 0 |
| rs12638540 | G | CMTM7 | 3p22.3 | A/G | 1 | 1 | 0 | 1 | 1 | 0 |
| rs12646447 | C |  | 4q25 | C/T | 0 | 0 | 0 | 0 | 0 | 0 |
| rs12725198 | A | TMEM82 | 1p36.21 | A/G | 1 | 1 | 0 | 1 | 1 | 0 |
| rs12733856 | A |  | 1q41 | A/C | 1 | 1 | 0 | 1 | 1 | 0 |
| rs12740374 | T | PSRC1 | 1p13.3 | G/T | 0 | 0 | 0 | 0 | 0 | 0 |
| rs127430 | G |  | 20q13.32 | A/G | 1 | 1 | 0 | 1 | 1 | 0 |
| rs13015955 | A |  | 2p24.1 | A/G | 1 | 1 | 0 | 1 | 1 | 0 |
| rs13083990 | T | CASR | 3q21.1 | C/T | 1 | 1 | 0 | 1 | 1 | 0 |
| rs13107325 | T | SLC39A8 | 4q24 | C/T | 1 | 1 | 0 | 1 | 1 | 0 |
| rs13161895 | T | RNF130 | 5q35.3 | C/T | 1 | 1 | 0 | 1 | 1 | 0 |
| rs13226650 | A | MLXIPL | 7q11.23 | A/G | 1 | 1 | 0 | 0 | 0 | 0 |
| rs13232179 | A | CRYGN | 7q36.1 | A/T | 0 | 0 | 0 | 0 | 0 | 0 |
| rs1332844 | T | PHACTR1 | 6p24.1 | C/T | 0 | 0 | 0 | 0 | 0 | 0 |
| rs1333047 | T | CDKN2BAS | 9p21.3 | A/T | 0 | 0 | 0 | 0 | 0 | 0 |
| rs1333049 | C | CDKN2BAS | 9p21.3 | C/G | 0 | 0 | 0 | 0 | 0 | 0 |
| rs13333226 | A | UMOD | 16p12.3 | A/G | 1 | 1 | 0 | 1 | 1 | 0 |
| rs13376333 | T | KCNN3 | 1q21.3 | C/T | 1 | 1 | 0 | 1 | 1 | 0 |
| rs13407662 | T |  | 2p16.2 | C/T | 1 | 1 | 0 | 1 | 1 | 0 |
| rs1364044 | A | ADAMTS12 | 5p13.2 | C/T | 1 | 1 | 0 | 1 | 1 | 0 |
| rs1387153 | C |  | 11q14.3 | C/T | 1 | 1 | 0 | 1 | 1 | 0 |
| rs1412444 | T | LIPA | 10q23.31 | A/G | 1 | 1 | 1 | 1 | 1 | 1 |
| rs1441756 | G |  | 8p21.3 | G/T | 1 | 0 | 0 | 1 | 0 | 0 |
| rs1445021 | A |  | 15q23 | C/T | 1 | 1 | 0 | 0 | 0 | 0 |
| rs1462872 | A | NRG1 | 8p12 | C/T | 1 | 1 | 0 | 1 | 1 | 0 |
| rs1465330 | A | ZDHHC22 | 14q24.3 | A/T | 0 | 0 | 0 | 0 | 0 | 0 |
| rs1466535 | C | LRP1 | 12q13.3 | C/T | 1 | 1 | 0 | 1 | 1 | 0 |
| rs1475591 | T |  | 21q22.11 | C/T | 1 | 1 | 0 | 0 | 0 | 0 |
| rs1526687 | T |  | 2p16.3 | G/T | 1 | 1 | 0 | 1 | 1 | 0 |
| rs1532085 | G |  | 15q21.3 | A/G | 0 | 0 | 0 | 0 | 0 | 0 |
| rs1535 | G | FADS2 | 11q12.2 | A/G | 0 | 0 | 0 | 0 | 0 | 0 |
| rs1537370 | T | CDKN2BAS | 9p21.3 | C/T | 0 | 0 | 0 | 0 | 0 | 0 |
| rs157582 | T | TOMM40 | 19q13.32 | A/G | 1 | 0 | 0 | 1 | 0 | 0 |
| rs161802 | T | PARK7 | 1p36.23 | G/T | 0 | 0 | 0 | 0 | 0 | 0 |
| rs16893526 | G |  | 6q14.1 | A/G | 1 | 1 | 0 | 1 | 1 | 0 |
| rs16965039 | T | NLRC5 | 16q13 | C/T | 1 | 1 | 0 | 0 | 0 | 0 |
| rs16971384 | A | ZFHX3 | 16q22.3 | A/G | 1 | 1 | 0 | 1 | 1 | 0 |
| rs16976171 | T |  | 18q12.3 | G/T | 1 | 1 | 0 | 1 | 1 | 0 |
| rs16982520 | A | ZNF831 | 20q13.32 | A/G | 1 | 1 | 0 | 1 | 1 | 0 |
| rs17015535 | A | WDR33 | 2q14.3 | A/T | 0 | 0 | 0 | 0 | 0 | 0 |
| rs17042171 | A |  | 4q25 | A/C | 0 | 0 | 0 | 0 | 0 | 0 |
| rs17045031 | A |  | 3p14.1 | A/G | 1 | 1 | 0 | 1 | 1 | 0 |
| rs17088339 | A |  | 18q22.3 | A/T | 0 | 0 | 0 | 0 | 0 | 0 |
| rs17091905 | A |  | 8p21.3 | A/G | 0 | 0 | 0 | 0 | 0 | 0 |
| rs17159640 | T | IFRD1 | 7q31.1 | A/T | 0 | 0 | 0 | 0 | 0 | 0 |
| rs17167021 | G |  | 5q21.1 | A/G | 1 | 1 | 0 | 0 | 0 | 0 |
| rs172166 | C |  | 6p22.1 | C/G | 0 | 0 | 0 | 0 | 0 | 0 |
| rs17228212 | C | SMAD3 | 15q22.33 | C/T | 1 | 1 | 0 | 1 | 1 | 0 |
| rs1735151 | T | IGSF5 | 21q22.2 | C/T | 1 | 1 | 0 | 1 | 1 | 0 |
| rs17375901 | T | MTHFR | 1p36.22 | C/T | 1 | 1 | 0 | 1 | 1 | 0 |
| rs17398575 | A |  | 7q22.3 | A/G | 1 | 1 | 0 | 0 | 0 | 0 |
| rs174546 | A | FADS1 | 11q12.2 | C/T | 1 | 1 | 0 | 1 | 1 | 0 |
| rs1746048 | C |  | 10q11.21 | C/T | 1 | 1 | 1 | 1 | 1 | 1 |
| rs17465637 | C | MIA3 | 1q41 | A/C | 1 | 1 | 1 | 0 | 0 | 0 |
| rs17609940 | G | ANKS1A | 6p21.31 | C/G | 0 | 0 | 0 | 0 | 0 | 0 |
| rs17666963 | T |  | 10q26.13 | C/T | 1 | 1 | 0 | 1 | 1 | 0 |
| rs17672135 | T | FMN2 | 1q43 | C/T | 1 | 1 | 0 | 1 | 1 | 0 |
| rs17696736 | G | NAA25 | 12q24.13 | A/G | 1 | 0 | 0 | 0 | 0 | 0 |
| rs17724172 | C | DLGAP1 | 18p11.31 | C/T | 1 | 1 | 0 | 0 | 0 | 0 |
| rs17772222 | A |  | 14q31.3 | A/G | 1 | 1 | 0 | 1 | 1 | 0 |
| rs1799945 | G | HFE | 6p22.2 | C/G | 0 | 0 | 0 | 0 | 0 | 0 |
| rs1800562 | A | HFE | 6p22.2 | A/G | 1 | 1 | 0 | 1 | 1 | 0 |
| rs1803274 | T | BCHE | 3q26.1 | A/G | 1 | 1 | 0 | 1 | 1 | 0 |
| rs1842896 | T |  | 4q32.1 | G/T | 1 | 1 | 0 | 1 | 1 | 0 |
| rs1878406 | T | EDNRA | 4q31.22 | C/T | 1 | 1 | 0 | 1 | 1 | 0 |
| rs1883025 | T | ABCA1 | 9q31.1 | A/G | 1 | 1 | 0 | 1 | 1 | 0 |
| rs1902341 | G | OSBPL10 | 3p23 | A/G | 1 | 1 | 0 | 1 | 1 | 0 |
| rs1937579 | T |  | 6q25.2 | A/G | 1 | 1 | 0 | 1 | 1 | 0 |
| rs1993293 | A |  | 15q26.3 | A/G | 1 | 1 | 0 | 0 | 0 | 0 |
| rs1994016 | C | ADAMTS7 | 15q25.1 | C/T | 0 | 0 | 0 | 0 | 0 | 0 |
| rs2040862 | T | WNT8A | 5q31.2 | C/T | 1 | 1 | 0 | 1 | 1 | 0 |
| rs2043085 | A |  | 15q21.3 | A/G | 1 | 1 | 0 | 1 | 1 | 0 |
| rs2048327 | C | SLC22A3 | 6q25.3 | A/G | 1 | 1 | 0 | 1 | 1 | 0 |
| rs2075290 | C | ZNF259 | 11q23.3 | C/T | 0 | 0 | 0 | 0 | 0 | 0 |
| rs2075650 | G | TOMM40 | 19q13.32 | A/G | 1 | 1 | 0 | 1 | 1 | 0 |
| rs2080401 | A |  | 2q31.1 | G/T | 1 | 1 | 0 | 1 | 1 | 0 |
| rs2083637 | G |  | 8p21.3 | C/T | 0 | 0 | 0 | 0 | 0 | 0 |
| rs2084898 | A |  | 11q23.3 | A/G | 1 | 1 | 0 | 1 | 1 | 0 |
| rs2106261 | T | ZFHX3 | 16q22.3 | A/G | 0 | 0 | 0 | 0 | 0 | 0 |
| rs2107595 | A |  | 7p21.1 | C/T | 1 | 0 | 0 | 1 | 0 | 0 |
| rs2123536 | T |  | 2p24.1 | A/G | 1 | 1 | 0 | 1 | 1 | 0 |
| rs2125623 | C | OTUD7A | 15q13.3 | C/T | 1 | 1 | 0 | 1 | 1 | 0 |
| rs216172 | C | SMG6 | 17p13.3 | C/G | 0 | 0 | 0 | 0 | 0 | 0 |
| rs2197089 | C | LPL | 8p21.3 | C/T | 1 | 0 | 0 | 1 | 0 | 0 |
| rs2199936 | A |  |  |  | 0 | 0 | 0 | 0 | 0 | 0 |
| rs2200733 | T |  | 4q25 | C/T | 1 | 0 | 0 | 1 | 0 | 0 |
| rs2201728 | G |  | 4q23 | A/G | 1 | 1 | 0 | 1 | 1 | 0 |
| rs2206277 | A | TFAP2B | 6p12.3 | A/G | 1 | 1 | 0 | 1 | 1 | 0 |
| rs2238151 | T | ALDH2 | 12q24.12 | C/T | 1 | 0 | 0 | 1 | 0 | 0 |
| rs225132 | G | ERRFI1 | 1p36.23 | A/C | 1 | 1 | 0 | 1 | 1 | 0 |
| rs2259816 | T | HNF1A | 12q24.31 | A/C | 1 | 1 | 1 | 1 | 1 | 1 |
| rs2266788 | C | APOA5 | 11q23.3 | C/T | 0 | 0 | 0 | 0 | 0 | 0 |
| rs2281727 | G | SMG6 | 17p13.3 | C/T | 0 | 0 | 0 | 0 | 0 | 0 |
| rs2306374 | C | MRAS | 3q22.3 | C/T | 0 | 0 | 0 | 0 | 0 | 0 |
| rs2332267 | T |  | 1q25.3 | A/G | 1 | 1 | 0 | 1 | 1 | 0 |
| rs2341260 | T |  | 1p31.1 | C/T | 1 | 1 | 0 | 1 | 1 | 0 |
| rs2346177 | A |  | 2p21 | A/G | 1 | 1 | 0 | 1 | 1 | 0 |
| rs2359536 | C |  | 10p12.31 | C/T | 1 | 1 | 0 | 1 | 1 | 0 |
| rs2383207 | G | CDKN2BAS | 9p21.3 | A/G | 0 | 0 | 0 | 0 | 0 | 0 |
| rs2398162 | A |  | 15q26.2 | A/G | 1 | 1 | 0 | 1 | 1 | 0 |
| rs2437258 | T |  |  |  | 0 | 0 | 0 | 0 | 0 | 0 |
| rs247616 | T | CETP | 16q13 | C/T | 1 | 1 | 0 | 1 | 1 | 0 |
| rs247617 | C | CETP | 16q13 | A/C | 0 | 0 | 0 | 0 | 0 | 0 |
| rs2505083 | C | KIAA1462 | 10p11.23 | C/T | 1 | 1 | 1 | 1 | 1 | 1 |
| rs2515629 | A | ABCA1 | 9q31.1 | C/T | 1 | 1 | 0 | 1 | 1 | 0 |
| rs2521501 | T | FES | 15q26.1 | A/T | 0 | 0 | 0 | 0 | 0 | 0 |
| rs2622633 | A | ZFPM2 | 8q23.1 | A/G | 1 | 1 | 0 | 0 | 0 | 0 |
| rs264 | A | LPL | 8p21.3 | A/G | 1 | 1 | 0 | 1 | 1 | 0 |
| rs2679073 | A |  | 15q25.3 | C/T | 1 | 1 | 0 | 1 | 1 | 0 |
| rs268 | G | LPL | 8p21.3 | A/G | 1 | 1 | 0 | 0 | 0 | 0 |
| rs2681472 | A | ATP2B1 | 12q21.33 | C/T | 1 | 1 | 0 | 1 | 1 | 0 |
| rs2820037 | T |  | 1q43 | A/T | 0 | 0 | 0 | 0 | 0 | 0 |
| rs2895811 | C | HHIPL1 | 14q32.2 | C/T | 1 | 1 | 1 | 1 | 1 | 1 |
| rs290227 | G | SYK | 9q22.2 | C/T | 1 | 1 | 0 | 1 | 1 | 0 |
| rs2932538 | G | MOV10 | 1p13.2 | C/T | 1 | 1 | 0 | 1 | 1 | 0 |
| rs2943634 | C |  | 2q36.3 | A/C | 1 | 1 | 0 | 1 | 1 | 0 |
| rs295 | A | LPL | 8p21.3 | A/C | 0 | 0 | 0 | 0 | 0 | 0 |
| rs301 | C | LPL | 8p21.3 | C/T | 0 | 0 | 0 | 0 | 0 | 0 |
| rs3099844 | A |  |  |  | 1 | 1 | 0 | 0 | 0 | 0 |
| rs3184504 | T | SH2B3 | 12q24.12 | C/T | 1 | 1 | 1 | 1 | 1 | 1 |
| rs34014631 | A | C10orf76 | 10q24.32 | A/G | 1 | 1 | 0 | 1 | 1 | 0 |
| rs365302 | C | FNDC1 | 6q25.3 | A/G | 1 | 1 | 0 | 1 | 1 | 0 |
| rs3729639 | T | E2F4 | 16q22.1 | C/T | 1 | 1 | 0 | 1 | 1 | 0 |
| rs3739998 | C | KIAA1462 | 10p11.23 | C/G | 0 | 0 | 0 | 0 | 0 | 0 |
| rs3742207 | C | COL4A1 | 13q34 | A/C | 1 | 1 | 0 | 1 | 1 | 0 |
| rs3757840 | A | YKT6 | 7p13 | A/C | 1 | 1 | 0 | 1 | 1 | 0 |
| rs3764261 | A | CETP | 16q13 | G/T | 0 | 0 | 0 | 0 | 0 | 0 |
| rs3782889 | C | MYL2 | 12q24.11 | C/T | 1 | 1 | 0 | 1 | 1 | 0 |
| rs3798220 | C | LPA | 6q25.3 | C/T | 1 | 1 | 1 | 1 | 1 | 1 |
| rs3807989 | G | CAV1 | 7q31.2 | A/G | 1 | 1 | 0 | 1 | 1 | 0 |
| rs3809346 | A | COL4A1 | 13q34 | C/T | 0 | 0 | 0 | 0 | 0 | 0 |
| rs3869109 | G |  |  |  | 1 | 1 | 0 | 0 | 0 | 0 |
| rs3894944 | A |  | 4p16.1 | C/T | 1 | 1 | 0 | 0 | 0 | 0 |
| rs3903239 | G |  | 1q24.2 | C/T | 1 | 1 | 0 | 1 | 1 | 0 |
| rs4304924 | G | RNF219 | 13q31.1 | A/G | 1 | 1 | 0 | 0 | 0 | 0 |
| rs4373814 | G | CACNB2 | 10p12.33 | C/G | 0 | 0 | 0 | 0 | 0 | 0 |
| rs4380028 | C | ADAMTS7 | 15q25.1 | A/G | 1 | 0 | 0 | 1 | 0 | 0 |
| rs4528684 | T |  | 19p13.12 | C/T | 1 | 1 | 0 | 1 | 1 | 0 |
| rs4635554 | G |  | 2p24.1 | G/T | 1 | 1 | 0 | 1 | 1 | 0 |
| rs46522 | T | UBE2Z | 17q21.32 | C/T | 1 | 1 | 1 | 1 | 1 | 1 |
| rs4665058 | A | BAZ2B | 2q24.2 | A/C | 1 | 1 | 0 | 1 | 1 | 0 |
| rs469568 | T | ADAMTS2 | 5q35.3 | G/T | 1 | 1 | 0 | 1 | 1 | 0 |
| rs4698036 | G |  | 4p16.1 | G/T | 1 | 0 | 0 | 1 | 0 | 0 |
| rs4712972 | A | SLC17A4 | 6p22.2 | A/G | 1 | 1 | 0 | 1 | 1 | 0 |
| rs4773144 | G | COL4A1 | 13q34 | A/G | 1 | 1 | 1 | 1 | 1 | 1 |
| rs4792143 | T | SHISA6 | 17p12 | C/T | 1 | 1 | 0 | 1 | 1 | 0 |
| rs4804155 | C | DOCK6 | 19p13.2 | C/G | 0 | 0 | 0 | 0 | 0 | 0 |
| rs4820834 | A | CCDC157 | 22q12.2 | A/G | 1 | 1 | 0 | 1 | 1 | 0 |
| rs4846922 | T | GALNT2 | 1q42.13 | C/T | 1 | 1 | 0 | 1 | 1 | 0 |
| rs4875320 | A | CSMD1 | 8p23.2 | A/T | 0 | 0 | 0 | 0 | 0 | 0 |
| rs4900022 | A | KCNK13 | 14q32.11 | A/G | 1 | 1 | 0 | 0 | 0 | 0 |
| rs4937126 | G | ST3GAL4 | 11q24.2 | A/G | 1 | 1 | 0 | 1 | 1 | 0 |
| rs496300 | G |  | 21q22.3 | A/G | 1 | 1 | 0 | 1 | 1 | 0 |
| rs4977574 | G | CDKN2BAS | 9p21.3 | A/G | 1 | 1 | 1 | 1 | 1 | 1 |
| rs4979906 | G |  | 10q22.3 | A/G | 1 | 1 | 0 | 1 | 1 | 0 |
| rs508487 | T | TAGLN | 11q23.3 | A/G | 1 | 0 | 0 | 1 | 0 | 0 |
| rs514659 | C | ABO | 9q34.2 | A/C | 1 | 0 | 0 | 1 | 0 | 0 |
| rs556621 | A |  | 6p21.1 | A/C | 1 | 1 | 0 | 1 | 1 | 0 |
| rs560887 | G | G6PC2 | 2q31.1 | A/G | 1 | 0 | 0 | 1 | 0 | 0 |
| rs569805 | A | ABCB11 | 2q31.1 | A/T | 0 | 0 | 0 | 0 | 0 | 0 |
| rs5754891 | A |  | 22q12.3 | A/G | 1 | 1 | 0 | 1 | 1 | 0 |
| rs579459 | C | ABO | 9q34.2 | C/T | 1 | 1 | 1 | 1 | 1 | 1 |
| rs5904726 | A | MIR508 | Xq27.3 | A/G | 0 | 0 | 0 | 0 | 0 | 0 |
| rs599839 | A | PSRC1 | 1p13.3 | A/G | 0 | 0 | 0 | 0 | 0 | 0 |
| rs6015450 | G |  | 20q13.32 | A/G | 0 | 0 | 0 | 0 | 0 | 0 |
| rs602633 | T | PSRC1 | 1p13.3 | A/C | 0 | 0 | 0 | 0 | 0 | 0 |
| rs605920 | C |  | 18q22.1 | C/G | 0 | 0 | 0 | 0 | 0 | 0 |
| rs632728 | T |  | 6p21.1 | A/G | 0 | 0 | 0 | 0 | 0 | 0 |
| rs633185 | G | ARHGAP42 | 11q22.1 | C/G | 0 | 0 | 0 | 0 | 0 | 0 |
| rs646776 | T | PSRC1 | 1p13.3 | A/G | 1 | 1 | 1 | 1 | 1 | 1 |
| rs6511720 | T | LDLR | 19p13.2 | G/T | 1 | 1 | 0 | 1 | 1 | 0 |
| rs6596140 | T |  | 5q31.1 | C/T | 1 | 1 | 0 | 1 | 1 | 0 |
| rs6601299 | T |  | 8p23.1 | C/T | 0 | 0 | 0 | 0 | 0 | 0 |
| rs6601530 | G | PINX1 | 8p23.1 | A/G | 1 | 1 | 0 | 1 | 1 | 0 |
| rs660240 | T | PSRC1 | 1p13.3 | A/G | 0 | 0 | 0 | 0 | 0 | 0 |
| rs6666258 | C | KCNN3 | 1q21.3 | C/G | 0 | 0 | 0 | 0 | 0 | 0 |
| rs671 | A | ALDH2 | 12q24.12 | A/G | 1 | 1 | 0 | 0 | 0 | 0 |
| rs6711016 | C |  | 2p24.1 | A/C | 1 | 0 | 0 | 1 | 0 | 0 |
| rs6725887 | C | WDR12 | 2q33.2 | C/T | 1 | 1 | 1 | 1 | 1 | 1 |
| rs673548 | G | APOB | 2p24.1 | A/G | 1 | 1 | 0 | 1 | 1 | 0 |
| rs6782380 | C |  | 3p14.3 | C/G | 0 | 0 | 0 | 0 | 0 | 0 |
| rs6817105 | C |  | 4q25 | C/T | 0 | 0 | 0 | 0 | 0 | 0 |
| rs6843082 | G |  | 4q25 | A/G | 1 | 1 | 0 | 1 | 1 | 0 |
| rs6868223 | A | ADAMTS12 | 5p13.3 | A/G | 1 | 1 | 0 | 1 | 1 | 0 |
| rs688034 | T | SEZ6L | 22q12.1 | C/T | 1 | 1 | 0 | 1 | 1 | 0 |
| rs6905288 | T | VEGFA | 6p21.1 | A/G | 1 | 1 | 0 | 0 | 0 | 0 |
| rs6922269 | A | MTHFD1L | 6q25.1 | A/G | 1 | 1 | 0 | 1 | 1 | 0 |
| rs6929568 | T |  | 6p24.3 | G/T | 1 | 1 | 0 | 1 | 1 | 0 |
| rs6947830 | A |  | 7p21.2 | A/G | 1 | 1 | 0 | 1 | 1 | 0 |
| rs6983473 | A | C8orf80 | 8p21.1 | A/T | 0 | 0 | 0 | 0 | 0 | 0 |
| rs7025486 | A | DAB2IP | 9q33.2 | A/G | 1 | 1 | 0 | 1 | 1 | 0 |
| rs7070038 | A |  | 10q21.1 | A/G | 1 | 1 | 0 | 1 | 1 | 0 |
| rs7092929 | A |  | 10p15.2 | A/C | 1 | 1 | 0 | 1 | 1 | 0 |
| rs7120489 | A | PARVA | 11p15.3 | A/G | 1 | 1 | 0 | 1 | 1 | 0 |
| rs7121446 | G | LOC399959 | 11q24.1 | A/G | 1 | 1 | 0 | 1 | 1 | 0 |
| rs7136259 | T |  | 12q21.33 | C/T | 1 | 1 | 0 | 1 | 1 | 0 |
| rs7164883 | G | HCN4 | 15q24.1 | A/G | 1 | 1 | 0 | 1 | 1 | 0 |
| rs7173743 | C |  | 15q25.1 | C/T | 1 | 0 | 0 | 1 | 0 | 0 |
| rs7193343 | T | ZFHX3 | 16q22.3 | C/T | 1 | 0 | 0 | 1 | 0 | 0 |
| rs7203193 | A | LITAF | 16p13.13 | A/G | 1 | 1 | 0 | 1 | 1 | 0 |
| rs7246657 | T | LOC284412 | 19q13.12 | C/T | 1 | 1 | 0 | 1 | 1 | 0 |
| rs7323893 | T |  | 13q31.2 | C/T | 0 | 0 | 0 | 0 | 0 | 0 |
| rs741013 | A |  | 3p14.1 | A/T | 0 | 0 | 0 | 0 | 0 | 0 |
| rs744487 | A |  | 1q42.3 | G/T | 1 | 1 | 0 | 1 | 1 | 0 |
| rs74733271 | A | PMAIP1 | 18q21.32 | G/T | 0 | 0 | 0 | 0 | 0 | 0 |
| rs749924 | T | LOC728323 | 2q37.3 | C/T | 1 | 1 | 0 | 0 | 0 | 0 |
| rs7550636 | T |  | 1q31.2 | C/T | 1 | 1 | 0 | 0 | 0 | 0 |
| rs7569328 | T |  | 2p24.1 | C/T | 1 | 1 | 0 | 1 | 1 | 0 |
| rs7581224 | T |  | 2p11.2 | G/T | 1 | 1 | 0 | 1 | 1 | 0 |
| rs7586540 | A |  | 2p25.2 | A/G | 1 | 1 | 0 | 1 | 1 | 0 |
| rs7651039 | C | BTD | 3p25.1 | C/T | 1 | 1 | 0 | 1 | 1 | 0 |
| rs7671266 | T |  | 4p16.1 | C/T | 1 | 1 | 0 | 1 | 1 | 0 |
| rs7687921 | T | GPM6A | 4q34.2 | A/T | 0 | 0 | 0 | 0 | 0 | 0 |
| rs7765175 | T |  | 6q21 | C/T | 1 | 1 | 0 | 1 | 1 | 0 |
| rs780094 | A | GCKR | 2p23.3 | A/G | 0 | 0 | 0 | 0 | 0 | 0 |
| rs7801190 | C | SLC12A9 | 7q22.1 | C/G | 0 | 0 | 0 | 0 | 0 | 0 |
| rs7808424 | G | ASZ1 | 7q31.2 | G/T | 1 | 1 | 0 | 1 | 1 | 0 |
| rs782590 | T | SMEK2 | 2p16.1 | C/T | 1 | 1 | 0 | 1 | 1 | 0 |
| rs7841189 | C |  | 8p21.3 | C/T | 1 | 0 | 0 | 1 | 0 | 0 |
| rs7856675 | A | SLC1A1 | 9p24.2 | A/G | 1 | 1 | 0 | 0 | 0 | 0 |
| rs7865146 | A | AK1 | 9q34.11 | C/T | 1 | 1 | 0 | 1 | 1 | 0 |
| rs7865618 | A | CDKN2BAS | 9p21.3 | A/G | 1 | 0 | 0 | 1 | 0 | 0 |
| rs7890572 | G | IL1RAPL1 | Xp21.2 | A/G | 0 | 0 | 0 | 0 | 0 | 0 |
| rs7903146 | A | TCF7L2 | 10q25.2 | C/T | 1 | 1 | 0 | 1 | 1 | 0 |
| rs7937106 | C |  | 11q22.3 | C/T | 1 | 1 | 0 | 1 | 1 | 0 |
| rs7965445 | A |  | 12q24.33 | A/G | 1 | 1 | 0 | 1 | 1 | 0 |
| rs8017423 | T |  | 14q32.11 | C/T | 1 | 1 | 0 | 0 | 0 | 0 |
| rs8028579 | T | SEMA6D | 15q21.1 | G/T | 0 | 0 | 0 | 0 | 0 | 0 |
| rs805303 | G |  |  |  | 1 | 1 | 0 | 0 | 0 | 0 |
| rs8055236 | G | CDH13 | 16q23.3 | G/T | 1 | 1 | 0 | 1 | 1 | 0 |
| rs8060686 | T | EDC4 | 16q22.1 | C/T | 1 | 1 | 0 | 1 | 1 | 0 |
| rs879324 | A | ZFHX3 | 16q22.3 | C/T | 1 | 1 | 0 | 1 | 1 | 0 |
| rs886126 | T | CUX2 | 12q24.11 | C/T | 1 | 1 | 0 | 1 | 1 | 0 |
| rs899435 | A |  | 12p13.1 | G/T | 1 | 1 | 0 | 1 | 1 | 0 |
| rs899967 | C | BCL2 | 18q21.33 | C/G | 0 | 0 | 0 | 0 | 0 | 0 |
| rs9268402 | G |  |  |  | 1 | 1 | 0 | 0 | 0 | 0 |
| rs9321637 | C |  | 6q23.3 | C/T | 1 | 1 | 0 | 1 | 1 | 0 |
| rs9326246 | C | BUD13 | 11q23.3 | C/G | 0 | 0 | 0 | 0 | 0 | 0 |
| rs932764 | G | PLCE1 | 10q23.33 | A/G | 1 | 1 | 0 | 1 | 1 | 0 |
| rs9328448 | A |  | 6p24.3 | A/G | 1 | 1 | 0 | 1 | 1 | 0 |
| rs9369640 | C | PHACTR1 | 6p24.1 | A/C | 0 | 0 | 0 | 0 | 0 | 0 |
| rs937254 | A | GCOM1 | 15q21.3 | A/G | 1 | 1 | 0 | 1 | 1 | 0 |
| rs9388451 | C | HEY2 | 6q22.31 | C/T | 1 | 1 | 0 | 1 | 1 | 0 |
| rs944797 | C | CDKN2BAS | 9p21.3 | C/T | 0 | 0 | 0 | 0 | 0 | 0 |
| rs9506514 | A | IFT88 | 13q12.11 | A/C | 1 | 1 | 0 | 1 | 1 | 0 |
| rs9508025 | C | FLT1 | 13q12.3 | C/G | 0 | 0 | 0 | 0 | 0 | 0 |
| rs9546711 | A |  | 13q31.1 | A/G | 1 | 1 | 0 | 1 | 1 | 0 |
| rs964184 | G | ZNF259 | 11q23.3 | C/G | 0 | 0 | 0 | 0 | 0 | 0 |
| rs974819 | T |  | 11q22.3 | A/G | 1 | 1 | 1 | 1 | 1 | 1 |
| rs978152 | C | KCNQ3 | 8q24.22 | C/G | 0 | 0 | 0 | 0 | 0 | 0 |
| rs9818870 | T | MRAS | 3q22.3 | C/T | 1 | 1 | 1 | 1 | 1 | 1 |
| rs9907236 | A |  | 17q24.3 | A/G | 1 | 1 | 0 | 1 | 1 | 0 |
| rs991316 | T |  | 4q23 | A/G | 1 | 1 | 0 | 1 | 1 | 0 |
| rs9939224 | G | CETP | 16q13 | G/T | 1 | 1 | 0 | 1 | 1 | 0 |
| rs9940128 | A | FTO | 16q12.2 | A/G | 1 | 1 | 0 | 1 | 1 | 0 |
| rs9982601 | T |  | 21q22.11 | C/T | 1 | 1 | 1 | 1 | 1 | 1 |
| rs9987289 | A |  | 8p23.1 | A/G | 1 | 1 | 0 | 1 | 1 | 0 |
| rs3825807 | A | ADAMTS7 | 15q25.1 | C/T | 1 | 1 | 1 | 0 | 0 | 0 |

**Table S3**

| **SNP** | **Risk Allele** | **Gene** | **Band** | **Alleles** | **In GRS1** | **In GRS2** | **In GRS3** | **In GRS4** | **In GRS5** | **In GRS6** |
| --- | --- | --- | --- | --- | --- | --- | --- | --- | --- | --- |
| rs10199521 | T |  | 2p25.3 | A/G | 1 | 1 | 0 | 1 | 1 | 0 |
| rs10229583 | G | PAX4 | 7q32.1 | A/G | 1 | 1 | 0 | 1 | 1 | 0 |
| rs10403021 | C |  | 19q12 | C/T | 1 | 1 | 0 | 1 | 1 | 0 |
| rs10440833 | A | CDKAL1 | 6p22.3 | A/T | 0 | 0 | 0 | 0 | 0 | 0 |
| rs10460009 | C | LOC727896 | 18p11.31 | C/T | 1 | 1 | 0 | 1 | 1 | 0 |
| rs10461617 | A | MAP3K1 | 5q11.2 | A/G | 1 | 1 | 0 | 1 | 1 | 0 |
| rs1048886 | G | C6orf57 | 6q13 | A/G | 1 | 1 | 0 | 1 | 1 | 0 |
| rs10520514 | A |  | 4q34.3 | A/T | 0 | 0 | 0 | 0 | 0 | 0 |
| rs1073203 | G |  | 5q23.2 | C/G | 0 | 0 | 0 | 0 | 0 | 0 |
| rs10741243 | G | TCERG1L | 10q26.3 | C/G | 0 | 0 | 0 | 0 | 0 | 0 |
| rs10744625 | C | EFCAB4B | 12p13.32 | C/T | 1 | 1 | 0 | 0 | 0 | 0 |
| rs10811661 | T |  | 9p21.3 | C/T | 1 | 1 | 1 | 1 | 1 | 1 |
| rs10814916 | C | GLIS3 | 9p24.2 | A/C | 1 | 1 | 0 | 1 | 1 | 0 |
| rs10830963 | G | MTNR1B | 11q14.3 | C/G | 0 | 0 | 0 | 0 | 0 | 0 |
| rs10885122 | G |  | 10q25.2 | G/T | 1 | 1 | 0 | 1 | 1 | 0 |
| rs10886471 | C | GRK5 | 10q26.11 | C/T | 1 | 1 | 0 | 1 | 1 | 0 |
| rs10906115 | A |  | 10p13 | A/G | 1 | 0 | 0 | 1 | 0 | 0 |
| rs10910200 | G |  | 1q42.2 | C/G | 0 | 0 | 0 | 0 | 0 | 0 |
| rs10923931 | T | NOTCH2 | 1p12 | G/T | 1 | 1 | 1 | 1 | 1 | 1 |
| rs10927101 | A |  | 1q44 | A/C | 1 | 0 | 0 | 1 | 0 | 0 |
| rs10946398 | C | CDKAL1 | 6p22.3 | A/C | 0 | 0 | 0 | 0 | 0 | 0 |
| rs10965250 | G |  | 9p21.3 | A/G | 0 | 0 | 0 | 0 | 0 | 0 |
| rs11071657 | A |  | 15q22.2 | A/G | 1 | 0 | 0 | 1 | 0 | 0 |
| rs1111875 | C | HHEX | 10q23.33 | A/G | 1 | 1 | 1 | 1 | 1 | 1 |
| rs11165354 | A | TGFBR3 | 1p22.1 | A/C | 1 | 1 | 0 | 1 | 1 | 0 |
| rs11257655 | T |  | 10p13 | C/T | 1 | 0 | 0 | 1 | 0 | 0 |
| rs1153188 | A |  | 12q13.2 | A/T | 0 | 0 | 0 | 0 | 0 | 0 |
| rs11558471 | A | SLC30A8 | 8q24.11 | A/G | 0 | 0 | 0 | 0 | 0 | 0 |
| rs11605924 | A | CRY2 | 11p11.2 | A/C | 1 | 1 | 0 | 1 | 1 | 0 |
| rs11634397 | G | ZFAND6 | 15q25.1 | A/G | 1 | 1 | 0 | 1 | 1 | 0 |
| rs11642841 | A | FTO | 16q12.2 | A/C | 0 | 0 | 0 | 0 | 0 | 0 |
| rs11677370 | T |  | 2p25.3 | A/T | 0 | 0 | 0 | 0 | 0 | 0 |
| rs11708067 | A | ADCY5 | 3q21.1 | A/G | 1 | 1 | 0 | 1 | 1 | 0 |
| rs11765845 | A | CREB5 | 7p15.1 | A/G | 1 | 1 | 0 | 1 | 1 | 0 |
| rs11787792 | A | GPSM1 | 9q34.3 | A/G | 1 | 1 | 0 | 0 | 0 | 0 |
| rs11867934 | C |  | 17p11.2 | C/T | 1 | 1 | 0 | 0 | 0 | 0 |
| rs11920090 | T | SLC2A2 | 3q26.2 | A/T | 0 | 0 | 0 | 0 | 0 | 0 |
| rs12010175 | G | FAM58A | Xq28 | A/G | 0 | 0 | 0 | 0 | 0 | 0 |
| rs12027542 | A | PCNXL2 | 1q42.2 | A/G | 1 | 1 | 0 | 1 | 1 | 0 |
| rs12219125 | T |  | 10p12.31 | G/T | 1 | 1 | 0 | 1 | 1 | 0 |
| rs12304921 | G | HIGD1C | 12q13.12 | A/G | 1 | 1 | 0 | 1 | 1 | 0 |
| rs12518099 | C |  | 5q14.3 | A/G | 1 | 1 | 0 | 1 | 1 | 0 |
| rs12655917 | C | AP3B1 | 5q14.1 | A/G | 1 | 1 | 0 | 0 | 0 | 0 |
| rs12779790 | G |  | 10p13 | A/G | 1 | 1 | 1 | 1 | 1 | 1 |
| rs12853515 | G | ARHGEF7 | 13q34 | C/G | 0 | 0 | 0 | 0 | 0 | 0 |
| rs12914656 | C |  | 15q25.3 | C/T | 1 | 1 | 0 | 1 | 1 | 0 |
| rs13064954 | G |  | 3q25.31 | A/G | 1 | 1 | 0 | 1 | 1 | 0 |
| rs13081389 | A |  | 3p25.2 | A/G | 1 | 0 | 0 | 1 | 0 | 0 |
| rs13266634 | C | SLC30A8 | 8q24.11 | C/T | 1 | 1 | 1 | 1 | 1 | 1 |
| rs13292136 | C |  | 9q21.31 | C/T | 1 | 1 | 0 | 1 | 1 | 0 |
| rs1333051 | A |  | 9p21.3 | A/T | 0 | 0 | 0 | 0 | 0 | 0 |
| rs1342038 | G |  | 1q25.1 | C/T | 1 | 1 | 0 | 1 | 1 | 0 |
| rs1359790 | G |  | 13q31.1 | C/T | 1 | 1 | 0 | 1 | 1 | 0 |
| rs1374910 | T | IGF2BP2 | 3q27.2 | C/T | 1 | 1 | 0 | 1 | 1 | 0 |
| rs1387153 | T |  | 11q14.3 | C/T | 1 | 0 | 0 | 1 | 0 | 0 |
| rs1401492 | C | CACNA1D | 3p21.1 | A/G | 1 | 1 | 0 | 1 | 1 | 0 |
| rs1436953 | G |  | 15q22.2 | A/G | 1 | 1 | 0 | 1 | 1 | 0 |
| rs1436955 | C |  | 15q22.2 | A/G | 1 | 0 | 0 | 1 | 0 | 0 |
| rs1470579 | C | IGF2BP2 | 3q27.2 | A/C | 1 | 1 | 1 | 1 | 1 | 1 |
| rs1495377 | G |  | 12q21.1 | C/G | 0 | 0 | 0 | 0 | 0 | 0 |
| rs1531343 | C | RPSAP52 | 12q14.3 | C/G | 0 | 0 | 0 | 0 | 0 | 0 |
| rs1552224 | A | ARAP1 | 11q13.4 | G/T | 1 | 1 | 0 | 1 | 1 | 0 |
| rs1571942 | C | PLXDC2 | 10p12.31 | C/T | 0 | 0 | 0 | 0 | 0 | 0 |
| rs163182 | C | KCNQ1 | 11p15.4 | C/G | 0 | 0 | 0 | 0 | 0 | 0 |
| rs16861329 | G | ST6GAL1 | 3q27.3 | C/T | 1 | 1 | 0 | 1 | 1 | 0 |
| rs16891077 | A | ISCA1P1 | 5q12.1 | A/G | 1 | 1 | 0 | 1 | 1 | 0 |
| rs16955379 | C | CMIP | 16q23.2 | C/T | 1 | 1 | 0 | 1 | 1 | 0 |
| rs16962638 | C |  | 13q33.1 | A/G | 0 | 0 | 0 | 0 | 0 | 0 |
| rs17036101 | G |  | 3p25.2 | A/G | 0 | 0 | 0 | 0 | 0 | 0 |
| rs17045328 | G | CR2 | 1q32.2 | A/G | 1 | 1 | 0 | 1 | 1 | 0 |
| rs17053082 | T |  | 5q33.2 | C/T | 1 | 1 | 0 | 1 | 1 | 0 |
| rs17376456 | A | C5orf36 | 5q15 | A/G | 1 | 1 | 0 | 1 | 1 | 0 |
| rs17404956 | A |  | 5q34 | A/G | 1 | 1 | 0 | 0 | 0 | 0 |
| rs17431357 | C | TRIAP1 | 12q24.31 | C/T | 1 | 1 | 0 | 1 | 1 | 0 |
| rs174550 | T | FADS1 | 11q12.2 | C/T | 1 | 1 | 0 | 1 | 1 | 0 |
| rs17584499 | T | PTPRD | 9p24.1 | C/T | 1 | 1 | 0 | 0 | 0 | 0 |
| rs17589516 | C | ZFAND3 | 6p21.2 | A/G | 1 | 1 | 0 | 1 | 1 | 0 |
| rs17797882 | T |  | 16q23.2 | C/T | 1 | 1 | 0 | 1 | 1 | 0 |
| rs1801214 | T | WFS1 | 4p16.1 | C/T | 0 | 0 | 0 | 0 | 0 | 0 |
| rs1801282 | C | PPARG | 3p25.2 | C/G | 0 | 0 | 0 | 0 | 0 | 0 |
| rs1802295 | A | SUPV3L1 | 10q22.1 | C/T | 1 | 1 | 0 | 1 | 1 | 0 |
| rs1861612 | T | DNER | 2q36.3 | A/G | 1 | 1 | 0 | 1 | 1 | 0 |
| rs1970671 | G |  | 18q21.2 | C/G | 0 | 0 | 0 | 0 | 0 | 0 |
| rs2028299 | C | AP3S2 | 15q26.1 | A/C | 1 | 1 | 0 | 1 | 1 | 0 |
| rs2038823 | C | HS6ST3 | 13q32.1 | A/C | 1 | 1 | 0 | 1 | 1 | 0 |
| rs2063640 | A | ZPLD1 | 3q12.3 | A/C | 1 | 1 | 0 | 1 | 1 | 0 |
| rs2115386 | C | INSR | 19p13.2 | A/G | 1 | 1 | 0 | 1 | 1 | 0 |
| rs2166706 | G |  | 11q14.3 | C/T | 1 | 0 | 0 | 1 | 0 | 0 |
| rs2191349 | T |  | 7p21.2 | G/T | 1 | 1 | 0 | 1 | 1 | 0 |
| rs2237892 | C | KCNQ1 | 11p15.4 | C/T | 1 | 1 | 0 | 1 | 1 | 0 |
| rs2237895 | C | KCNQ1 | 11p15.4 | A/C | 1 | 1 | 0 | 1 | 1 | 0 |
| rs2237897 | C | KCNQ1 | 11p15.4 | C/T | 1 | 1 | 0 | 0 | 0 | 0 |
| rs231362 | G | KCNQ1OT1 | 11p15.5 | C/T | 1 | 1 | 0 | 1 | 1 | 0 |
| rs2383208 | A |  | 9p21.3 | A/G | 0 | 0 | 0 | 0 | 0 | 0 |
| rs2407103 | C |  | 8p11.23 | A/G | 1 | 1 | 0 | 1 | 1 | 0 |
| rs2407314 | C | CSMD1 | 8p23.2 | C/G | 0 | 0 | 0 | 0 | 0 | 0 |
| rs243021 | A |  | 2p16.1 | C/T | 1 | 0 | 0 | 1 | 0 | 0 |
| rs2696835 | C | LOC732275 | 16q24.1 | C/G | 0 | 0 | 0 | 0 | 0 | 0 |
| rs2722769 | C |  | 11p15.3 | C/G | 0 | 0 | 0 | 0 | 0 | 0 |
| rs2811893 | T | MYSM1 | 1p32.1 | C/T | 1 | 1 | 0 | 1 | 1 | 0 |
| rs2833610 | A | HUNK | 21q22.11 | A/G | 1 | 1 | 0 | 1 | 1 | 0 |
| rs2943641 | C |  | 2q36.3 | C/T | 1 | 1 | 0 | 1 | 1 | 0 |
| rs3007729 | T |  | 1p36.13 | C/T | 1 | 1 | 0 | 1 | 1 | 0 |
| rs30360 | G |  | 5q11.2 | A/C | 1 | 1 | 0 | 1 | 1 | 0 |
| rs312457 | G | SLC16A13 | 17p13.1 | C/T | 1 | 1 | 0 | 0 | 0 | 0 |
| rs328506 | C |  | 20q13.31 | C/T | 1 | 1 | 0 | 1 | 1 | 0 |
| rs340874 | C | PROX1 | 1q32.3 | A/G | 1 | 1 | 0 | 1 | 1 | 0 |
| rs35767 | G | IGF1 | 12q23.2 | C/T | 1 | 1 | 0 | 1 | 1 | 0 |
| rs3773506 | C | PLS1 | 3q23 | C/G | 0 | 0 | 0 | 0 | 0 | 0 |
| rs3786897 | A | PEPD | 19q13.11 | A/G | 1 | 1 | 0 | 1 | 1 | 0 |
| rs3792615 | T | 1-Mar | 4q32.3 | A/G | 1 | 1 | 0 | 1 | 1 | 0 |
| rs3802177 | G | SLC30A8 | 8q24.11 | C/T | 0 | 0 | 0 | 0 | 0 | 0 |
| rs391300 | G | SRR | 17p13.3 | A/G | 1 | 1 | 0 | 1 | 1 | 0 |
| rs3916765 | A |  |  |  | 1 | 1 | 0 | 0 | 0 | 0 |
| rs3923113 | A |  | 2q24.3 | G/T | 1 | 1 | 0 | 1 | 1 | 0 |
| rs4402960 | T | IGF2BP2 | 3q27.2 | G/T | 0 | 0 | 0 | 0 | 0 | 0 |
| rs4430796 | G | HNF1B | 17q12 | A/G | 1 | 1 | 0 | 0 | 0 | 0 |
| rs4457053 | G | LOC728723 | 5q13.3 | A/G | 1 | 1 | 0 | 1 | 1 | 0 |
| rs4462262 | C |  | 10q21.1 | C/T | 1 | 1 | 0 | 1 | 1 | 0 |
| rs4470583 | A |  | 4q32.2 | A/G | 1 | 1 | 0 | 0 | 0 | 0 |
| rs4506565 | T | TCF7L2 | 10q25.2 | A/T | 0 | 0 | 0 | 0 | 0 | 0 |
| rs4527850 | T | WISP1 | 8q24.22 | C/T | 1 | 1 | 0 | 0 | 0 | 0 |
| rs4607103 | C | MIR548A2 | 3p14.1 | C/T | 1 | 1 | 1 | 1 | 1 | 1 |
| rs4689388 | T | WFS1 | 4p16.1 | A/G | 1 | 1 | 0 | 1 | 1 | 0 |
| rs4698790 | T | GAR1 | 4q25 | G/T | 1 | 1 | 0 | 1 | 1 | 0 |
| rs4712523 | G | CDKAL1 | 6p22.3 | A/G | 0 | 0 | 0 | 0 | 0 | 0 |
| rs4712524 | G | CDKAL1 | 6p22.3 | A/G | 0 | 0 | 0 | 0 | 0 | 0 |
| rs472265 | G | PAPL | 19q13.2 | A/G | 1 | 1 | 0 | 1 | 1 | 0 |
| rs4760790 | A |  | 12q21.1 | A/G | 0 | 0 | 0 | 0 | 0 | 0 |
| rs476141 | A |  | 1q44 | A/C | 1 | 1 | 0 | 1 | 1 | 0 |
| rs4787008 | G | A2BP1 | 16p13.3 | A/G | 1 | 1 | 0 | 1 | 1 | 0 |
| rs4812829 | A | R3HDML | 20q13.12 | A/G | 1 | 1 | 0 | 1 | 1 | 0 |
| rs4819143 | C |  | 21q22.3 | A/G | 1 | 1 | 0 | 1 | 1 | 0 |
| rs4838605 | C | ARHGAP22 | 10q11.22 | C/T | 1 | 1 | 0 | 1 | 1 | 0 |
| rs5015480 | C |  | 10q23.33 | C/T | 0 | 0 | 0 | 0 | 0 | 0 |
| rs5215 | C | KCNJ11 | 11p15.1 | C/T | 0 | 0 | 0 | 0 | 0 | 0 |
| rs563694 | C | G6PC2 | 2q31.1 | G/T | 1 | 1 | 0 | 1 | 1 | 0 |
| rs564398 | T | CDKN2BAS | 9p21.3 | A/G | 1 | 1 | 0 | 1 | 1 | 0 |
| rs591044 | A | SEZ6L | 22q12.1 | C/T | 1 | 1 | 0 | 1 | 1 | 0 |
| rs5945326 | A | DUSP9 | Xq28 | A/G | 0 | 0 | 0 | 0 | 0 | 0 |
| rs6017317 | G | FITM2 | 20q13.12 | G/T | 1 | 0 | 0 | 1 | 0 | 0 |
| rs623323 | T | RNMTL1 | 17p13.3 | A/G | 1 | 1 | 0 | 1 | 1 | 0 |
| rs6426514 | A | RHOU | 1q42.13 | A/G | 1 | 1 | 0 | 1 | 1 | 0 |
| rs642858 | A |  | 6q24.1 | C/T | 1 | 1 | 0 | 1 | 1 | 0 |
| rs6467136 | G |  | 7q32.1 | A/G | 1 | 1 | 0 | 1 | 1 | 0 |
| rs649891 | C | PTPRD | 9p23 | A/G | 1 | 1 | 0 | 1 | 1 | 0 |
| rs6576507 | A |  | 15q12 | C/T | 1 | 1 | 0 | 0 | 0 | 0 |
| rs6583826 | G | KIF11 | 10q23.33 | A/G | 1 | 0 | 0 | 1 | 0 | 0 |
| rs6702784 | C | OSCP1 | 1p34.3 | A/C | 1 | 1 | 0 | 1 | 1 | 0 |
| rs6707387 | A | SPAG16 | 2q34 | A/G | 1 | 1 | 0 | 1 | 1 | 0 |
| rs6723108 | T | TMEM163 | 2q21.3 | G/T | 1 | 1 | 0 | 1 | 1 | 0 |
| rs6769511 | C | IGF2BP2 | 3q27.2 | C/T | 0 | 0 | 0 | 0 | 0 | 0 |
| rs6780569 | G |  | 3p24.3 | A/G | 1 | 1 | 0 | 1 | 1 | 0 |
| rs6931514 | G | CDKAL1 | 6p22.3 | A/G | 0 | 0 | 0 | 0 | 0 | 0 |
| rs7020996 | C | CDKN2BAS | 9p21.3 | C/T | 1 | 0 | 0 | 0 | 0 | 0 |
| rs7034200 | A | GLIS3 | 9p24.2 | A/C | 0 | 0 | 0 | 0 | 0 | 0 |
| rs7041847 | A | GLIS3 | 9p24.2 | A/G | 0 | 0 | 0 | 0 | 0 | 0 |
| rs7043482 | G |  | 9q21.32 | A/C | 1 | 1 | 0 | 1 | 1 | 0 |
| rs7107217 | C |  | 11q24.3 | A/C | 1 | 1 | 0 | 1 | 1 | 0 |
| rs7119 | T | HMG20A | 15q24.3 | A/G | 1 | 1 | 0 | 1 | 1 | 0 |
| rs7172432 | A |  | 15q22.2 | A/G | 1 | 0 | 0 | 1 | 0 | 0 |
| rs7178572 | G | HMG20A | 15q24.3 | A/G | 1 | 0 | 0 | 1 | 0 | 0 |
| rs7305618 | C | C12orf27 | 12q24.31 | C/T | 1 | 1 | 0 | 1 | 1 | 0 |
| rs730570 | G | C14orf70 | 14q32.2 | A/G | 1 | 1 | 0 | 1 | 1 | 0 |
| rs7403531 | T | RASGRP1 | 15q14 | C/T | 1 | 1 | 0 | 1 | 1 | 0 |
| rs742731 | A |  | 20p11.23 | A/G | 1 | 1 | 0 | 1 | 1 | 0 |
| rs7542900 | C |  | 1p21.3 | C/T | 1 | 1 | 0 | 1 | 1 | 0 |
| rs7560163 | C |  | 2q23.3 | C/G | 0 | 0 | 0 | 0 | 0 | 0 |
| rs7578326 | A |  | 2q36.3 | A/G | 1 | 0 | 0 | 1 | 0 | 0 |
| rs7578597 | T | THADA | 2p21 | C/T | 1 | 1 | 1 | 1 | 1 | 1 |
| rs7630877 | A | PEX5L | 3q26.33 | A/G | 1 | 1 | 0 | 1 | 1 | 0 |
| rs7636 | A | ACHE | 7q22.1 | C/T | 1 | 1 | 0 | 1 | 1 | 0 |
| rs7659604 | T |  | 4q27 | C/T | 1 | 1 | 0 | 1 | 1 | 0 |
| rs7754840 | C | CDKAL1 | 6p22.3 | C/G | 0 | 0 | 0 | 0 | 0 | 0 |
| rs7756992 | G | CDKAL1 | 6p22.3 | A/G | 0 | 0 | 0 | 0 | 0 | 0 |
| rs7766070 | A | CDKAL1 | 6p22.3 | A/C | 1 | 0 | 0 | 1 | 0 | 0 |
| rs7772697 | T |  | 6q25.1 | C/T | 1 | 1 | 0 | 1 | 1 | 0 |
| rs780094 | C | GCKR | 2p23.3 | A/G | 1 | 1 | 0 | 1 | 1 | 0 |
| rs7901695 | C | TCF7L2 | 10q25.2 | C/T | 0 | 0 | 0 | 0 | 0 | 0 |
| rs7903146 | A | TCF7L2 | 10q25.2 | C/T | 1 | 1 | 1 | 1 | 1 | 1 |
| rs791595 | A |  | 7q32.1 | A/G | 1 | 1 | 0 | 1 | 1 | 0 |
| rs7944584 | A | MADD | 11p11.2 | A/T | 0 | 0 | 0 | 0 | 0 | 0 |
| rs7957197 | T | OASL | 12q24.31 | A/T | 0 | 0 | 0 | 0 | 0 | 0 |
| rs7961581 | C |  | 12q21.1 | C/T | 1 | 1 | 1 | 1 | 1 | 1 |
| rs8042680 | A | PRC1 | 15q26.1 | A/C | 1 | 1 | 0 | 1 | 1 | 0 |
| rs8048589 | C | SNX29 | 16p13.13 | C/T | 1 | 1 | 0 | 1 | 1 | 0 |
| rs8050136 | A | FTO | 16q12.2 | A/C | 0 | 0 | 0 | 0 | 0 | 0 |
| rs8090011 | G | LAMA1 | 18p11.31 | C/G | 0 | 0 | 0 | 0 | 0 | 0 |
| rs8181588 | A | KCNQ1 | 11p15.4 | C/T | 1 | 0 | 0 | 1 | 0 | 0 |
| rs831571 | c |  | 3p14.1 | C/T | 1 | 1 | 0 | 1 | 1 | 0 |
| rs849134 | A | JAZF1 | 7p15.1 | A/G | 0 | 0 | 0 | 0 | 0 | 0 |
| rs864745 | T | JAZF1 | 7p15.1 | A/G | 1 | 1 | 1 | 1 | 1 | 1 |
| rs896854 | T | TP53INP1 | 8q22.1 | A/G | 1 | 1 | 0 | 1 | 1 | 0 |
| rs9295474 | G | CDKAL1 | 6p22.3 | C/G | 0 | 0 | 0 | 0 | 0 | 0 |
| rs9300039 | C |  | 11p12 | A/C | 1 | 1 | 0 | 1 | 1 | 0 |
| rs9391988 | A | SLC22A23 | 6p25.2 | A/G | 1 | 1 | 0 | 1 | 1 | 0 |
| rs9465871 | C | CDKAL1 | 6p22.3 | C/T | 1 | 0 | 0 | 1 | 0 | 0 |
| rs9470794 | C | ZFAND3 | 6p21.2 | C/T | 1 | 1 | 0 | 1 | 1 | 0 |
| rs9472138 | T |  | 6p21.1 | C/T | 1 | 1 | 1 | 1 | 1 | 1 |
| rs9525916 | T |  | 13q14.11 | A/T | 0 | 0 | 0 | 0 | 0 | 0 |
| rs9543976 | G | UCHL3 | 13q22.2 | A/G | 1 | 1 | 0 | 1 | 1 | 0 |
| rs9552911 | G | SGCG | 13q12.12 | A/G | 1 | 1 | 0 | 0 | 0 | 0 |
| rs972283 | G |  | 7q32.3 | A/G | 1 | 1 | 0 | 1 | 1 | 0 |
| rs9787485 | T |  | 10q23.1 | C/T | 1 | 1 | 0 | 1 | 1 | 0 |
| rs9792548 | C |  | 9q21.33 | A/G | 1 | 1 | 0 | 1 | 1 | 0 |
| rs9862730 | A | CLSTN2 | 3q23 | A/G | 1 | 1 | 0 | 1 | 1 | 0 |
| rs9866141 | T |  | 3q25.31 | C/T | 1 | 1 | 0 | 1 | 1 | 0 |
| rs9939609 | A | FTO | 16q12.2 | A/T | 0 | 0 | 0 | 0 | 0 | 0 |
| rs5219 | T |  | 11p15.1 | C/T | 1 | 1 | 1 | 1 | 1 | 1 |
| rs10490072 | T |  | 2p16.1 | C/T | 1 | 1 | 1 | 1 | 1 | 1 |

**Table S4**

| **SNP** | **Risk Allele** | **Gene** | **Band** | **Alleles** | **In GRS1** | **In GRS2** | **In GRS4** | **In GRS5** |
| --- | --- | --- | --- | --- | --- | --- | --- | --- |
| rs1000589 | T |  | 13q21.31 | G/T | 1 | 1 | 1 | 1 |
| rs10052657 | C | PDE4D | 5q11.2 | A/C | 1 | 1 | 1 | 1 |
| rs10058728 | T | CSNK1A1 | 5q32 | A/T | 0 | 0 | 0 | 0 |
| rs10069690 | T | TERT | 5p15.33 | C/T | 1 | 1 | 0 | 0 |
| rs10088218 | G |  | 8q24.21 | A/G | 1 | 1 | 1 | 1 |
| rs10088262 | A |  | 8q24.13 | A/G | 1 | 1 | 1 | 1 |
| rs10090154 | T |  | 8q24.21 | C/T | 0 | 0 | 0 | 0 |
| rs1011970 | T | CDKN2BAS | 9p21.3 | G/T | 1 | 1 | 1 | 1 |
| rs1014971 | T |  | 22q13.1 | A/G | 1 | 1 | 1 | 1 |
| rs1016343 | T |  | 8q24.21 | C/T | 1 | 1 | 1 | 1 |
| rs10170236 | C |  | 2q23.2 | A/G | 1 | 1 | 1 | 1 |
| rs10187424 | A | GGCX | 2p11.2 | C/T | 1 | 1 | 1 | 1 |
| rs10197940 | T |  | 2q23.3 | C/T | 1 | 1 | 1 | 1 |
| rs1027643 | T |  | 5q14.3 | C/T | 1 | 1 | 1 | 1 |
| rs103294 | C | LILRA3 | 19q13.42 | C/T | 1 | 1 | 1 | 1 |
| rs10411161 | T | ZNF577 | 19q13.41 | C/T | 1 | 1 | 1 | 1 |
| rs10411210 | C | RHPN2 | 19q13.11 | C/T | 1 | 1 | 1 | 1 |
| rs10419226 | A | CRTC1 | 19p13.11 | G/T | 1 | 1 | 1 | 1 |
| rs1044873 | C | IRF8 | 16q24.1 | C/T | 1 | 0 | 1 | 0 |
| rs10472076 | C |  | 5q11.2 | C/T | 1 | 1 | 1 | 1 |
| rs10484561 | G |  |  |  | 1 | 1 | 0 | 0 |
| rs10486567 | G | JAZF1 | 7p15.2 | A/G | 1 | 1 | 1 | 1 |
| rs10500715 | T | SBF2 | 11p15.4 | G/T | 1 | 1 | 1 | 1 |
| rs10503733 | T | NKX3-1 | 8p21.2 | A/C | 1 | 0 | 1 | 0 |
| rs10505477 | A |  | 8q24.21 | C/T | 0 | 0 | 0 | 0 |
| rs10505483 | T |  | 8q24.21 | A/G | 0 | 0 | 0 | 0 |
| rs10510102 | G | ATE1 | 10q26.13 | A/G | 1 | 1 | 1 | 1 |
| rs10759243 | A |  | 9q31.2 | A/C | 1 | 1 | 1 | 1 |
| rs10771399 | T |  | 12p11.22 | A/G | 1 | 1 | 1 | 1 |
| rs10774214 | T |  | 12p13.32 | C/T | 1 | 1 | 1 | 1 |
| rs10775480 | T | SLC14A1 | 18q12.3 | C/T | 1 | 0 | 1 | 0 |
| rs1078806 | C | FGFR2 | 10q26.13 | C/T | 0 | 0 | 0 | 0 |
| rs10788473 | T | GRID1 | 10q23.1 | C/T | 1 | 1 | 1 | 1 |
| rs10795668 | A |  | 10p14 | A/G | 1 | 1 | 1 | 1 |
| rs10821936 | C | ARID5B | 10q21.2 | C/T | 1 | 1 | 1 | 1 |
| rs10822013 | T | ZNF365 | 10q21.2 | C/T | 1 | 1 | 1 | 1 |
| rs10828317 | T | PIP4K2A | 10p12.2 | C/T | 1 | 1 | 1 | 1 |
| rs10849033 | G | C12orf5 | 12p13.32 | A/G | 1 | 1 | 0 | 0 |
| rs10873876 | T | ST6GALNAC3 | 1p31.1 | C/T | 1 | 1 | 1 | 1 |
| rs10875943 | C | TUBA1C | 12q13.12 | C/T | 1 | 1 | 1 | 1 |
| rs10896449 | G |  | 11q13.3 | A/G | 0 | 0 | 0 | 0 |
| rs10911251 | A | LAMC1 | 1q25.3 | A/C | 1 | 1 | 1 | 1 |
| rs1092913 | T | ROPN1L | 5p15.2 | A/G | 1 | 1 | 1 | 1 |
| rs10934853 | A | EEFSEC | 3q21.3 | A/C | 1 | 1 | 1 | 1 |
| rs10936599 | C | ARPM1 | 3q26.2 | C/T | 1 | 1 | 1 | 1 |
| rs10936632 | A | CLDN11 | 3q26.2 | A/C | 1 | 1 | 0 | 0 |
| rs10937405 | C | TP63 | 3q28 | C/T | 1 | 1 | 1 | 1 |
| rs10941679 | G |  | 5p12 | A/G | 1 | 0 | 1 | 0 |
| rs10953615 | C |  | 7q31.1 | A/G | 1 | 1 | 1 | 1 |
| rs10974531 | A |  | 9p24.2 | A/C | 1 | 1 | 1 | 1 |
| rs10993994 | T | MSMB | 10q11.23 | C/T | 1 | 1 | 1 | 1 |
| rs10995190 | G | ZNF365 | 10q21.2 | A/G | 1 | 1 | 1 | 1 |
| rs11012732 | A | MLLT10 | 10p12.31 | A/G | 1 | 1 | 1 | 1 |
| rs11022157 | A | C11orf21 | 11p15.5 | A/C | 1 | 1 | 0 | 0 |
| rs11066015 | A | ACAD10 | 12q24.12 | A/G | 0 | 0 | 0 | 0 |
| rs11066280 | A | C12orf51 | 12q24.13 | A/T | 0 | 0 | 0 | 0 |
| rs11080466 | C | FAM38B | 18p11.22 | A/G | 1 | 1 | 1 | 1 |
| rs11083846 | A | PRKD2 | 19q13.32 | A/G | 1 | 1 | 1 | 1 |
| rs11135910 | A | EBF2 | 8p21.2 | C/T | 1 | 1 | 1 | 1 |
| rs11155133 | G |  | 6q24.1 | A/G | 1 | 1 | 0 | 0 |
| rs11169552 | C | ATF1 | 12q13.12 | C/T | 1 | 1 | 1 | 1 |
| rs11196174 | G | TCF7L2 | 10q25.2 | A/G | 1 | 1 | 0 | 0 |
| rs11199914 | C |  | 10q26.12 | C/T | 1 | 1 | 0 | 0 |
| rs11228565 | A |  | 11q13.3 | A/G | 1 | 0 | 1 | 0 |
| rs11242675 | T | FOXQ1 | 6p25.3 | C/T | 1 | 1 | 1 | 1 |
| rs11249433 | G | LOC647121 | 1p11.2 | C/T | 1 | 1 | 1 | 1 |
| rs11552449 | T | DCLRE1B | 1p13.2 | C/T | 1 | 1 | 1 | 1 |
| rs11568818 | A | MMP7 | 11q22.2 | A/G | 1 | 1 | 1 | 1 |
| rs11571833 | T | N4BP2L1 | 13q13.1 | A/T | 0 | 0 | 0 | 0 |
| rs11636802 | G |  | 15q21.3 | A/G | 1 | 1 | 1 | 1 |
| rs11650494 | A |  | 17q21.32 | A/G | 1 | 1 | 1 | 1 |
| rs11672691 | G |  | 19q13.2 | A/G | 1 | 1 | 1 | 1 |
| rs116909374 | T |  |  |  | 1 | 1 | 0 | 0 |
| rs11704416 | C | TNRC6B | 22q13.1 | C/G | 0 | 0 | 0 | 0 |
| rs11780156 | T |  | 8q24.21 | C/T | 1 | 1 | 1 | 1 |
| rs11782652 | A | CHMP4C | 8q21.13 | A/G | 1 | 1 | 1 | 1 |
| rs11789015 | A | BARX1 | 9q22.32 | A/G | 1 | 1 | 1 | 1 |
| rs11814448 | C |  | 10p12.31 | A/C | 1 | 1 | 1 | 1 |
| rs11820646 | C |  | 11q24.3 | C/T | 0 | 0 | 0 | 0 |
| rs11892031 | A | UGT1A8 | 2q37.1 | A/C | 1 | 1 | 1 | 1 |
| rs11902236 | A | GRHL1 | 2p25.1 | C/T | 1 | 1 | 1 | 1 |
| rs11903757 | C |  | 2q32.3 | C/T | 1 | 1 | 0 | 0 |
| rs11978267 | G | IKZF1 | 7p12.2 | A/G | 0 | 0 | 0 | 0 |
| rs1209950 | T | ETS2 | 21q22.2 | C/T | 1 | 1 | 1 | 1 |
| rs12155172 | A |  | 7p15.3 | A/G | 1 | 1 | 1 | 1 |
| rs1218582 | G | KCNN3 | 1q21.3 | C/T | 1 | 1 | 1 | 1 |
| rs1219648 | G | FGFR2 | 10q26.13 | A/G | 0 | 0 | 0 | 0 |
| rs12202284 | A |  | 6p25.3 | A/C | 0 | 0 | 0 | 0 |
| rs12203592 | T | IRF4 | 6p25.3 | C/T | 1 | 0 | 0 | 0 |
| rs12210050 | T | EXOC2 | 6p25.3 | C/T | 0 | 0 | 0 | 0 |
| rs12289961 | T |  | 11q12.1 | C/T | 1 | 0 | 1 | 0 |
| rs12296850 | A | SLC17A8 | 12q23.1 | A/G | 1 | 1 | 0 | 0 |
| rs12355688 | T | ZMIZ1 | 10q22.3 | C/T | 1 | 1 | 0 | 0 |
| rs12413624 | T |  | 10q26.11 | A/T | 0 | 0 | 0 | 0 |
| rs12422552 | C |  | 12p13.1 | C/G | 0 | 0 | 0 | 0 |
| rs1243180 | T | MLLT10 | 10p12.31 | A/T | 0 | 0 | 0 | 0 |
| rs12456874 | G | C18orf1 | 18p11.21 | A/G | 1 | 1 | 0 | 0 |
| rs12493607 | C | TGFBR2 | 3p24.1 | C/G | 0 | 0 | 0 | 0 |
| rs12615966 | A |  | 2q12.1 | C/T | 1 | 1 | 1 | 1 |
| rs12621643 | T | KCNE4 | 2q36.1 | G/T | 1 | 1 | 1 | 1 |
| rs12653946 | T |  | 5p15.33 | C/T | 1 | 1 | 0 | 0 |
| rs1270884 | A |  | 12q24.21 | C/T | 1 | 1 | 1 | 1 |
| rs1292011 | A |  | 12q24.21 | C/T | 1 | 1 | 1 | 1 |
| rs12922061 | T |  | 16q12.2 | C/T | 1 | 0 | 1 | 0 |
| rs130067 | G |  |  |  | 1 | 1 | 0 | 0 |
| rs13016963 | A | ALS2CR12 | 2q33.1 | A/G | 1 | 1 | 1 | 1 |
| rs13117307 | T | EXOC1 | 4q12 | C/T | 1 | 1 | 1 | 1 |
| rs13181 | C | KLC3 | 19q13.32 | G/T | 1 | 1 | 1 | 1 |
| rs132390 | C | EMID1 | 22q12.2 | C/T | 1 | 1 | 0 | 0 |
| rs13252298 | A |  | 8q24.21 | A/G | 1 | 1 | 1 | 1 |
| rs13254738 | C |  | 8q24.21 | A/C | 1 | 1 | 1 | 1 |
| rs13281615 | G |  | 8q24.21 | A/G | 1 | 0 | 1 | 0 |
| rs13294589 | G |  | 9p21.2 | A/G | 1 | 1 | 1 | 1 |
| rs13329835 | G | CDYL2 | 16q23.2 | A/G | 1 | 1 | 1 | 1 |
| rs13385191 | G | C2orf43 | 2p24.1 | A/G | 1 | 1 | 1 | 1 |
| rs13387042 | A |  | 2q35 | A/G | 1 | 1 | 1 | 1 |
| rs13393577 | T | MIR548F2 | 2q34 | C/T | 1 | 1 | 1 | 1 |
| rs13397985 | G | SP140 | 2q37.1 | G/T | 1 | 1 | 1 | 1 |
| rs13401811 | G | ACOXL | 2q13 | A/G | 1 | 1 | 1 | 1 |
| rs1353747 | T | PDE4D | 5q11.2 | G/T | 1 | 1 | 1 | 1 |
| rs1393350 | A | TYR | 11q14.3 | A/G | 1 | 1 | 1 | 1 |
| rs1427593 | A |  | 2q22.1 | A/G | 1 | 1 | 1 | 1 |
| rs1432295 | G |  | 2p16.1 | A/G | 1 | 1 | 1 | 1 |
| rs1432679 | C | EBF1 | 5q33.3 | C/T | 1 | 1 | 1 | 1 |
| rs1436904 | T | CHST9 | 18q11.2 | G/T | 1 | 1 | 1 | 1 |
| rs1439287 | A | BCL2L11 | 2q13 | C/T | 1 | 1 | 1 | 1 |
| rs1447295 | A | LOC727677 | 8q24.21 | A/C | 0 | 0 | 0 | 0 |
| rs1494961 | C | MRPS18C | 4q21.23 | C/T | 1 | 1 | 1 | 1 |
| rs1495741 | A |  | 8p22 | A/G | 1 | 1 | 1 | 1 |
| rs1496766 | C | MAGI2 | 7q21.11 | C/T | 1 | 1 | 0 | 0 |
| rs1512268 | T | NKX3-1 | 8p21.2 | A/G | 1 | 1 | 1 | 1 |
| rs1547374 | A | TFF1 | 21q22.3 | A/G | 1 | 1 | 1 | 1 |
| rs1550623 | A | CDCA7 | 2q31.1 | C/T | 1 | 1 | 1 | 1 |
| rs1562430 | T |  | 8q24.21 | A/G | 1 | 0 | 1 | 0 |
| rs1585440 | C |  | 13q21.32 | A/C | 1 | 1 | 1 | 1 |
| rs1665650 | T | HSPA12A | 10q25.3 | A/G | 1 | 1 | 1 | 1 |
| rs1679013 | C |  | 9p21.3 | C/T | 1 | 1 | 0 | 0 |
| rs16857609 | T | DIRC3 | 2q35 | C/T | 1 | 0 | 1 | 0 |
| rs16886165 | G |  | 5q11.2 | G/T | 1 | 1 | 1 | 1 |
| rs16892766 | A |  | 8q23.3 | A/C | 1 | 1 | 1 | 1 |
| rs16901979 | A |  | 8q24.21 | A/C | 0 | 0 | 0 | 0 |
| rs16902094 | G |  | 8q24.21 | A/G | 1 | 1 | 1 | 1 |
| rs16951095 | C | LAMA1 | 18p11.31 | C/T | 1 | 1 | 0 | 0 |
| rs16953002 | A | FTO | 16q12.2 | A/G | 1 | 1 | 1 | 1 |
| rs16976734 | G | RFX7 | 15q21.3 | A/G | 1 | 0 | 1 | 0 |
| rs17023900 | G |  | 3p12.1 | A/G | 1 | 1 | 1 | 1 |
| rs17079534 | A | MYRIP | 3p22.1 | A/G | 0 | 0 | 0 | 0 |
| rs17172185 | T | HECW1 | 7p14.1 | C/T | 1 | 1 | 1 | 1 |
| rs17206779 | G | ADAMTS6 | 5q12.3 | C/T | 1 | 1 | 1 | 1 |
| rs17221259 | G |  | 12p13.1 | C/T | 1 | 1 | 1 | 1 |
| rs17246404 | C | POT1 | 7q31.33 | C/T | 1 | 1 | 1 | 1 |
| rs17356907 | A |  | 12q22 | A/G | 1 | 1 | 0 | 0 |
| rs17401966 | A | KIF1B | 1p36.22 | A/G | 1 | 1 | 1 | 1 |
| rs17483466 | G | ACOXL | 2q13 | A/G | 1 | 1 | 1 | 1 |
| rs17530068 | C |  | 6q14.1 | C/T | 1 | 1 | 1 | 1 |
| rs17631303 | G |  |  |  | 1 | 1 | 1 | 1 |
| rs17674580 | T | SLC14A1 | 18q12.3 | C/T | 1 | 1 | 1 | 1 |
| rs17761864 | A | SMG6 | 17p13.3 | A/C | 1 | 1 | 1 | 1 |
| rs17817449 | T | FTO | 16q12.2 | G/T | 1 | 1 | 1 | 1 |
| rs17837497 | A | TBXAS1 | 7q34 | A/G | 0 | 0 | 0 | 0 |
| rs1789924 | C | ADH1C | 4q23 | C/T | 1 | 1 | 1 | 1 |
| rs1800682 | A | FASAS | 10q23.31 | C/T | 1 | 0 | 1 | 0 |
| rs183211 | A | NSF | 17q21.31 | A/G | 1 | 1 | 1 | 1 |
| rs1859962 | G |  | 17q24.3 | G/T | 1 | 1 | 1 | 1 |
| rs1879352 | C |  | 18p11.32 | A/G | 1 | 1 | 1 | 1 |
| rs1881797 | C | LOC148824 | 1q44 | C/T | 1 | 1 | 1 | 1 |
| rs1886449 | A |  | 13q22.1 | C/T | 1 | 1 | 1 | 1 |
| rs1894292 | G | AFM | 4q13.3 | A/G | 1 | 1 | 1 | 1 |
| rs1906953 | A | GRM4 | 6p21.31 | C/T | 1 | 1 | 1 | 1 |
| rs1933488 | A | RGS17 | 6q25.2 | A/G | 1 | 1 | 1 | 1 |
| rs1983891 | T | FOXP4 | 6p21.1 | C/T | 1 | 1 | 1 | 1 |
| rs2014300 | G | RUNX1 | 21q22.12 | A/G | 1 | 1 | 1 | 1 |
| rs2016394 | G | DLX2 | 2q31.1 | C/T | 1 | 1 | 1 | 1 |
| rs2019960 | G |  | 8q24.21 | C/T | 1 | 1 | 1 | 1 |
| rs2039553 | A |  | 13q31.1 | A/G | 1 | 1 | 1 | 1 |
| rs2041840 | C | C2orf56 | 2p22.2 | A/G | 1 | 1 | 1 | 1 |
| rs204247 | G |  | 6p23 | A/G | 1 | 1 | 1 | 1 |
| rs2046210 | A | C6orf97 | 6q25.1 | C/T | 1 | 1 | 1 | 1 |
| rs2048672 | C | FLJ43663 | 7q32.3 | G/T | 1 | 1 | 1 | 1 |
| rs2072590 | T | HOXD3 | 2q31.1 | G/T | 1 | 1 | 1 | 1 |
| rs2074356 | T | C12orf51 | 12q24.13 | C/T | 0 | 0 | 0 | 0 |
| rs2086452 | C | ADAMTS17 | 15q26.3 | A/G | 1 | 1 | 0 | 0 |
| rs2089222 | A | MAP1LC3B2 | 12q24.22 | A/G | 1 | 1 | 1 | 1 |
| rs210134 | G | BAK1 | 6p21.31 | A/G | 0 | 0 | 0 | 0 |
| rs210142 | C | BAK1 | 6p21.31 | C/T | 1 | 1 | 1 | 1 |
| rs2121875 | G | FGF10 | 5p12 | G/T | 1 | 1 | 1 | 1 |
| rs2131877 | G | C3orf21 | 3q29 | C/T | 1 | 1 | 1 | 1 |
| rs2178146 | A |  | 16q24.1 | A/G | 1 | 1 | 1 | 1 |
| rs2180341 | G | RNF146 | 6q22.33 | A/G | 1 | 1 | 1 | 1 |
| rs2191566 | G | ZNF230 | 19q13.31 | A/C | 1 | 1 | 1 | 1 |
| rs2236007 | G | PAX9 | 14q13.3 | A/G | 1 | 1 | 1 | 1 |
| rs2236256 | C | OPRM1 | 6q25.2 | A/C | 1 | 1 | 1 | 1 |
| rs2239612 | T | ST6GAL1 | 3q27.3 | C/T | 1 | 1 | 1 | 1 |
| rs2239633 | G | CEBPE | 14q11.2 | C/T | 1 | 0 | 1 | 0 |
| rs2239815 | T | XBP1 | 22q12.1 | C/T | 1 | 0 | 1 | 0 |
| rs2242652 | G | TERT | 5p15.33 | C/T | 0 | 0 | 0 | 0 |
| rs224278 | C | EGR2 | 10q21.3 | C/T | 1 | 1 | 1 | 1 |
| rs225190 | G | MYO1D | 17q11.2 | A/G | 1 | 1 | 1 | 1 |
| rs2255280 | T | DAB2 | 5p13.1 | A/C | 1 | 1 | 0 | 0 |
| rs2257205 | A | RNF43 | 17q22 | A/G | 1 | 1 | 1 | 1 |
| rs2273669 | G | ARMC2 | 6q21 | A/G | 1 | 1 | 1 | 1 |
| rs2274223 | G | PLCE1 | 10q23.33 | A/G | 0 | 0 | 0 | 0 |
| rs2283873 | A | TCN2 | 22q12.2 | A/G | 1 | 1 | 0 | 0 |
| rs2285947 | A | DNAH11 | 7p15.3 | A/G | 1 | 1 | 1 | 1 |
| rs2290854 | A | MDM4 | 1q32.1 | C/T | 1 | 1 | 1 | 1 |
| rs2292884 | G | MLPH | 2q37.3 | A/G | 1 | 1 | 1 | 1 |
| rs2294008 | T | PSCA | 8q24.3 | C/T | 1 | 1 | 1 | 1 |
| rs2342002 | T |  | 6q11.1 | C/T | 1 | 1 | 0 | 0 |
| rs2352028 | A | GPC5 | 13q31.3 | C/T | 1 | 1 | 1 | 1 |
| rs2363956 | T | ANKLE1 | 19p13.11 | G/T | 0 | 0 | 0 | 0 |
| rs2371030 | G |  | 2q34 | A/G | 1 | 1 | 1 | 1 |
| rs2380205 | C |  | 10p15.1 | C/T | 1 | 1 | 1 | 1 |
| rs2395185 | T |  |  |  | 1 | 1 | 0 | 0 |
| rs2405942 | A | SHROOM2 | Xp22.2 | A/G | 0 | 0 | 0 | 0 |
| rs2423279 | C |  | 20p12.3 | C/T | 1 | 1 | 1 | 1 |
| rs2427345 | G |  | 20q13.33 | C/T | 1 | 1 | 1 | 1 |
| rs2439302 | G | NRG1 | 8p12 | C/G | 0 | 0 | 0 | 0 |
| rs2466024 | A |  | 8q24.21 | A/G | 1 | 0 | 1 | 0 |
| rs2466035 | C |  | 8q24.21 | C/T | 1 | 1 | 1 | 1 |
| rs2494938 | A | LRFN2 | 6p21.1 | A/G | 1 | 1 | 1 | 1 |
| rs2495478 | T | PCSK9 | 1p32.3 | C/T | 1 | 1 | 1 | 1 |
| rs2511714 | G | ODF1 | 8q22.3 | G/T | 1 | 1 | 1 | 1 |
| rs2562796 | T | HIBCH | 2q32.2 | G/T | 1 | 1 | 1 | 1 |
| rs258322 | A | CDK10 | 16q24.3 | C/T | 1 | 1 | 1 | 1 |
| rs2588809 | T | RAD51L1 | 14q24.1 | C/T | 1 | 1 | 1 | 1 |
| rs2596542 | A |  |  |  | 1 | 1 | 0 | 0 |
| rs2608053 | G | PVT1 | 8q24.21 | A/G | 1 | 1 | 1 | 1 |
| rs2621416 | G |  |  |  | 1 | 1 | 0 | 0 |
| rs2647012 | G |  |  |  | 1 | 1 | 0 | 0 |
| rs2660753 | T |  | 3p12.1 | C/T | 1 | 0 | 1 | 0 |
| rs2665390 | C | LOC100287227 | 3q25.31 | C/T | 1 | 1 | 1 | 1 |
| rs2687201 | T |  | 3p13 | A/C | 1 | 1 | 1 | 1 |
| rs2689154 | G |  | 1q43 | C/G | 0 | 0 | 0 | 0 |
| rs2735839 | G | KLK3 | 19q13.33 | A/G | 1 | 1 | 1 | 1 |
| rs2797501 | A | GDI2 | 10p15.1 | A/G | 1 | 1 | 1 | 1 |
| rs2808630 | G | CRP | 1q23.2 | C/T | 1 | 1 | 1 | 1 |
| rs2823093 | G |  | 21q21.1 | A/G | 1 | 1 | 1 | 1 |
| rs2847281 | C | PTPN2 | 18p11.21 | C/T | 1 | 1 | 1 | 1 |
| rs2853677 | C | TERT | 5p15.33 | C/T | 1 | 1 | 0 | 0 |
| rs2900333 | C | PLBD1 | 12p13.1 | C/T | 1 | 1 | 1 | 1 |
| rs2926702 | T | NCOA2 | 8q13.3 | A/G | 1 | 1 | 1 | 1 |
| rs2943559 | G |  | 8q21.11 | A/G | 1 | 1 | 1 | 1 |
| rs2981578 | C | FGFR2 | 10q26.13 | A/G | 1 | 0 | 1 | 0 |
| rs2981579 | A | FGFR2 | 10q26.13 | C/T | 1 | 1 | 1 | 1 |
| rs2981582 | A | FGFR2 | 10q26.13 | C/T | 0 | 0 | 0 | 0 |
| rs3016539 | A | PARK2 | 6q26 | A/G | 1 | 1 | 1 | 1 |
| rs3096702 | A |  |  |  | 1 | 1 | 0 | 0 |
| rs3112612 | T |  | 16q12.2 | C/T | 1 | 0 | 1 | 0 |
| rs3117582 | C |  |  |  | 1 | 1 | 0 | 0 |
| rs31489 | C | CLPTM1L | 5p15.33 | A/C | 0 | 0 | 0 | 0 |
| rs31490 | A | CLPTM1L | 5p15.33 | A/G | 1 | 0 | 1 | 0 |
| rs3217810 | T | CCND2 | 12p13.32 | C/T | 0 | 0 | 0 | 0 |
| rs3217901 | G | CCND2 | 12p13.32 | A/G | 1 | 1 | 1 | 1 |
| rs3219090 | C | PARP1 | 1q42.12 | A/G | 1 | 1 | 1 | 1 |
| rs339331 | T | RFX6 | 6q22.1 | C/T | 1 | 1 | 1 | 1 |
| rs34289250 | C | BRIP1 | 17q23.2 | C/T | 0 | 0 | 0 | 0 |
| rs36600 | A | MTMR3 | 22q12.2 | C/T | 1 | 1 | 1 | 1 |
| rs372883 | T | BACH1 | 21q21.3 | A/G | 1 | 1 | 1 | 1 |
| rs3734805 | C | C6orf97 | 6q25.1 | A/C | 1 | 0 | 1 | 0 |
| rs3750817 | T | FGFR2 | 10q26.13 | C/T | 1 | 0 | 1 | 0 |
| rs3755132 | G | DDX1 | 2p24.3 | G/T | 1 | 1 | 1 | 1 |
| rs3757318 | A | C6orf97 | 6q25.1 | A/G | 1 | 1 | 1 | 1 |
| rs3760982 | A | KCNN4 | 19q13.31 | A/G | 1 | 1 | 1 | 1 |
| rs3765524 | T | PLCE1 | 10q23.33 | C/T | 1 | 0 | 1 | 0 |
| rs3769825 | T | CASP8 | 2q33.1 | C/T | 1 | 1 | 1 | 1 |
| rs3771570 | A | FARP2 | 2q37.3 | C/T | 1 | 1 | 1 | 1 |
| rs3781264 | C | PLCE1 | 10q23.33 | C/T | 1 | 1 | 1 | 1 |
| rs3784099 | A | RAD51L1 | 14q24.1 | A/G | 1 | 1 | 1 | 1 |
| rs3790844 | T | NR5A2 | 1q32.1 | C/T | 1 | 1 | 1 | 1 |
| rs3802842 | C | C11orf93 | 11q23.1 | A/C | 1 | 1 | 1 | 1 |
| rs3803662 | A | TOX3 | 16q12.1 | C/T | 1 | 1 | 1 | 1 |
| rs3806624 | G | EOMES | 3p24.1 | C/T | 1 | 1 | 1 | 1 |
| rs3814113 | T |  | 9p22.2 | C/T | 1 | 1 | 1 | 1 |
| rs3817198 | C | LSP1 | 11p15.5 | C/T | 1 | 0 | 1 | 0 |
| rs3817963 | G |  |  |  | 1 | 1 | 0 | 0 |
| rs3824662 | T | GATA3 | 10p14 | G/T | 1 | 1 | 1 | 1 |
| rs3850699 | A | TRIM8 | 10q24.32 | C/T | 1 | 1 | 1 | 1 |
| rs3903072 | G |  | 11q13.1 | A/C | 1 | 1 | 1 | 1 |
| rs391023 | G | IRF8 | 16q24.1 | A/G | 1 | 1 | 1 | 1 |
| rs402710 | C | CLPTM1L | 5p15.33 | C/T | 1 | 1 | 1 | 1 |
| rs4072037 | A | MUC1 | 1q22 | A/G | 1 | 1 | 1 | 1 |
| rs41322152 | C | NPFFR1 | 10q22.1 | C/G | 0 | 0 | 0 | 0 |
| rs4132601 | C | IKZF1 | 7p12.2 | G/T | 1 | 1 | 1 | 1 |
| rs4242382 | A |  | 8q24.21 | A/G | 1 | 0 | 1 | 0 |
| rs4242384 | C |  | 8q24.21 | A/C | 1 | 1 | 1 | 1 |
| rs4245739 | A | MDM4 | 1q32.1 | A/C | 0 | 0 | 0 | 0 |
| rs4269383 | C |  | 6q25.3 | A/G | 1 | 1 | 1 | 1 |
| rs4322600 | G |  | 14q31.3 | A/G | 1 | 1 | 1 | 1 |
| rs4324798 | A |  |  |  | 1 | 1 | 0 | 0 |
| rs4368253 | C |  | 18q21.32 | C/T | 1 | 1 | 1 | 1 |
| rs4406737 | G | FAS | 10q23.31 | A/G | 1 | 1 | 1 | 1 |
| rs4415084 | T |  | 5p12 | C/T | 1 | 0 | 0 | 0 |
| rs4430796 | A | HNF1B | 17q12 | A/G | 1 | 0 | 0 | 0 |
| rs4444235 | C | BMP4 | 14q22.2 | C/T | 1 | 1 | 1 | 1 |
| rs445114 | T |  | 8q24.21 | C/T | 1 | 1 | 1 | 1 |
| rs4474514 | A | KITLG | 12q21.32 | A/G | 1 | 1 | 1 | 1 |
| rs4488809 | C | TP63 | 3q28 | C/T | 1 | 0 | 1 | 0 |
| rs4510656 | C | CDKAL1 | 6p22.3 | A/C | 1 | 1 | 1 | 1 |
| rs4530903 | T |  |  |  | 1 | 1 | 0 | 0 |
| rs455804 | C | NCRNA00110 | 21q21.3 | G/T | 1 | 1 | 1 | 1 |
| rs4610302 | G | SPARCL1 | 4q22.1 | A/G | 1 | 1 | 1 | 1 |
| rs4635969 | T | CLPTM1L | 5p15.33 | C/T | 1 | 1 | 1 | 1 |
| rs4691139 | G | TRIM61 | 4q32.3 | A/G | 1 | 1 | 1 | 1 |
| rs4767364 | A | NAA25 | 12q24.13 | A/G | 1 | 1 | 1 | 1 |
| rs4784227 | T |  | 16q12.1 | C/T | 0 | 0 | 0 | 0 |
| rs4785204 | T | HEATR3 | 16q12.1 | C/T | 1 | 1 | 1 | 1 |
| rs4785763 | A | DBNDD1 | 16q24.3 | A/C | 1 | 1 | 1 | 1 |
| rs4795519 | C |  | 17q11.1 | A/C | 1 | 1 | 1 | 1 |
| rs4808801 | A | ELL | 19p13.11 | A/G | 1 | 1 | 1 | 1 |
| rs4813802 | G |  | 20p12.3 | G/T | 1 | 1 | 1 | 1 |
| rs4822983 | T | CHEK2 | 22q12.1 | C/T | 0 | 0 | 0 | 0 |
| rs4849887 | C |  | 2q14.2 | C/T | 1 | 1 | 1 | 1 |
| rs4869742 | T | C6orf97 | 6q25.1 | C/T | 1 | 1 | 1 | 1 |
| rs4924410 | A | SRP14 | 15q15.1 | A/C | 1 | 1 | 1 | 1 |
| rs4924935 | G | PRPSAP2 | 17p11.2 | C/T | 1 | 1 | 1 | 1 |
| rs4925386 | C | LAMA5 | 20q13.33 | C/T | 1 | 1 | 1 | 1 |
| rs4927850 | A |  | 3q29 | C/T | 1 | 1 | 1 | 1 |
| rs4939827 | T | SMAD7 | 18q21.1 | C/T | 1 | 1 | 1 | 1 |
| rs4962416 | C | CTBP2 | 10q26.13 | C/T | 1 | 1 | 1 | 1 |
| rs4982731 | C | CEBPE | 14q11.2 | C/T | 1 | 1 | 1 | 1 |
| rs4987855 | G | BCL2 | 18q21.33 | A/G | 1 | 1 | 1 | 1 |
| rs501764 | C | FLJ45983 | 10p14 | G/T | 1 | 1 | 1 | 1 |
| rs505922 | C | ABO | 9q34.2 | C/T | 1 | 1 | 1 | 1 |
| rs527616 | G |  | 18q11.2 | C/G | 0 | 0 | 0 | 0 |
| rs539901 | G |  | 15q21.3 | G/T | 1 | 1 | 0 | 0 |
| rs546784 | A | PDE4B | 1p31.3 | A/G | 0 | 0 | 0 | 0 |
| rs563507 | A | PARD3 | 10p11.21 | A/G | 1 | 1 | 1 | 1 |
| rs573666 | G |  | 13q14.3 | A/G | 1 | 1 | 1 | 1 |
| rs5768709 | G | FAM19A5 | 22q13.32 | A/G | 1 | 1 | 1 | 1 |
| rs5919432 | A |  | Xq12 | C/T | 0 | 0 | 0 | 0 |
| rs59336 | T | TBX3 | 12q24.21 | A/T | 0 | 0 | 0 | 0 |
| rs5945572 | A | NUDT11 | Xp11.22 | A/G | 0 | 0 | 0 | 0 |
| rs5945619 | C | NUDT11 | Xp11.22 | C/T | 0 | 0 | 0 | 0 |
| rs5955543 | G | NHS | Xp22.13 | A/G | 0 | 0 | 0 | 0 |
| rs6001930 | C | MKL1 | 22q13.1 | C/T | 1 | 1 | 1 | 1 |
| rs6062509 | A | LIME1 | 20q13.33 | G/T | 1 | 1 | 1 | 1 |
| rs6104690 | A |  | 20p12.2 | A/G | 1 | 1 | 1 | 1 |
| rs614367 | T |  | 11q13.3 | C/T | 1 | 1 | 1 | 1 |
| rs616488 | A | PEX14 | 1p36.22 | C/T | 1 | 0 | 1 | 0 |
| rs6428370 | G |  | 1q31.3 | A/G | 1 | 1 | 1 | 1 |
| rs6457327 | C |  |  |  | 1 | 1 | 0 | 0 |
| rs6464375 | A | DPP6 | 7q36.2 | C/T | 1 | 1 | 1 | 1 |
| rs6465657 | C | LMTK2 | 7q21.3 | C/T | 1 | 1 | 1 | 1 |
| rs647161 | A |  | 5q31.1 | A/C | 1 | 1 | 1 | 1 |
| rs6472903 | T |  | 8q21.11 | G/T | 1 | 1 | 1 | 1 |
| rs6479272 | T | SLC35D2 | 9q22.32 | C/T | 1 | 1 | 1 | 1 |
| rs6479527 | C | PTPDC1 | 9q22.32 | A/G | 1 | 0 | 1 | 0 |
| rs6503659 | A | HAP1 | 17q21.2 | A/T | 0 | 0 | 0 | 0 |
| rs6504950 | G | STXBP4 | 17q22 | A/G | 1 | 1 | 1 | 1 |
| rs651164 | G | SLC22A1 | 6q25.3 | A/G | 1 | 1 | 1 | 1 |
| rs6683977 | C | PDE4B | 1p31.3 | C/G | 0 | 0 | 0 | 0 |
| rs6687758 | G |  | 1q41 | A/G | 1 | 1 | 1 | 1 |
| rs6691170 | T |  | 1q41 | G/T | 1 | 1 | 1 | 1 |
| rs671 | A | ALDH2 | 12q24.12 | A/G | 1 | 1 | 0 | 0 |
| rs6711606 | A | RNF149 | 2q11.2 | G/T | 1 | 1 | 1 | 1 |
| rs6736997 | A |  | 2q37.2 | A/C | 1 | 1 | 0 | 0 |
| rs674313 | T |  | 6p21.32 | A/G | 1 | 1 | 0 | 0 |
| rs6753473 | G | GPR113 | 2p23.3 | G/T | 1 | 1 | 1 | 1 |
| rs6759952 | T | DIRC3 | 2q35 | C/T | 1 | 0 | 1 | 0 |
| rs6762644 | G | ITPR1 | 3p26.1 | A/G | 1 | 1 | 1 | 1 |
| rs6763931 | T | ZBTB38 | 3q23 | A/G | 1 | 1 | 1 | 1 |
| rs6772209 | G |  | 3q26.31 | A/G | 0 | 0 | 0 | 0 |
| rs6773854 | C |  | 3q27.3 | C/T | 1 | 1 | 1 | 1 |
| rs6788895 | G | SIAH2 | 3q25.1 | G/T | 1 | 1 | 0 | 0 |
| rs6828523 | C | ADAM29 | 4q34.1 | A/C | 1 | 1 | 1 | 1 |
| rs684232 | G | VPS53 | 17p13.3 | A/G | 1 | 1 | 1 | 1 |
| rs6858698 | C | CAMK2D | 4q26 | C/G | 0 | 0 | 0 | 0 |
| rs6869388 | C | C5orf36 | 5q15 | C/T | 1 | 1 | 1 | 1 |
| rs6869841 | A |  | 5q35.2 | C/T | 1 | 1 | 1 | 1 |
| rs6879627 | G |  | 5p15.33 | C/T | 1 | 1 | 1 | 1 |
| rs6903608 | G |  |  |  | 1 | 1 | 0 | 0 |
| rs6964969 | C | IKZF1 | 7p12.2 | A/G | 0 | 0 | 0 | 0 |
| rs6983267 | G |  | 8q24.21 | G/T | 1 | 1 | 1 | 1 |
| rs6983561 | C |  | 8q24.21 | A/C | 1 | 1 | 0 | 0 |
| rs7014346 | A | POU5F1B | 8q24.21 | A/G | 1 | 0 | 1 | 0 |
| rs7023329 | A | MTAP | 9p21.3 | A/G | 1 | 1 | 1 | 1 |
| rs7040024 | A | DMRT1 | 9p24.3 | A/C | 1 | 1 | 1 | 1 |
| rs704010 | T | ZMIZ1 | 10q22.3 | A/G | 1 | 1 | 1 | 1 |
| rs7072776 | A | MLLT10 | 10p12.31 | A/G | 1 | 0 | 1 | 0 |
| rs707824 | T |  | 6p23 | A/G | 1 | 1 | 1 | 1 |
| rs708224 | A | BICD1 | 12p11.21 | A/G | 1 | 1 | 1 | 1 |
| rs7086803 | A | VTI1A | 10q25.2 | A/G | 1 | 1 | 1 | 1 |
| rs7089424 | C | ARID5B | 10q21.2 | G/T | 0 | 0 | 0 | 0 |
| rs710521 | A |  | 3q28 | A/G | 1 | 1 | 1 | 1 |
| rs7107217 | C |  | 11q24.3 | A/C | 1 | 1 | 1 | 1 |
| rs7130881 | G |  | 11q13.3 | A/G | 1 | 1 | 1 | 1 |
| rs7141529 | G |  | 14q24.1 | C/T | 1 | 1 | 1 | 1 |
| rs7142143 | C | PYGL | 14q22.1 | C/T | 1 | 1 | 0 | 0 |
| rs716274 | G |  | 11q22.3 | A/G | 1 | 1 | 1 | 1 |
| rs7176508 | A |  | 15q23 | A/G | 1 | 1 | 1 | 1 |
| rs720475 | G | ARHGEF5 | 7q35 | A/G | 1 | 1 | 1 | 1 |
| rs721048 | A | EHBP1 | 2p15 | A/G | 1 | 1 | 1 | 1 |
| rs7216064 | A | BPTF | 17q24.2 | A/G | 1 | 1 | 1 | 1 |
| rs7241993 | G |  | 18q23 | C/T | 1 | 1 | 1 | 1 |
| rs732765 | G | DLST | 14q24.3 | A/G | 1 | 1 | 1 | 1 |
| rs7335046 | G | UBAC2 | 13q32.3 | C/G | 0 | 0 | 0 | 0 |
| rs735665 | A |  | 11q24.1 | A/G | 1 | 1 | 1 | 1 |
| rs738722 | T | HSCB | 22q12.1 | C/T | 1 | 1 | 1 | 1 |
| rs7412746 | T |  | 1q21.3 | C/T | 1 | 1 | 1 | 1 |
| rs742134 | G | BIK | 22q13.2 | A/G | 1 | 1 | 1 | 1 |
| rs7501939 | C | HNF1B | 17q12 | C/T | 1 | 1 | 1 | 1 |
| rs7504990 | A | DCC | 18q21.2 | C/T | 1 | 1 | 1 | 1 |
| rs7538876 | A | PADI6 | 1p36.13 | A/G | 1 | 1 | 1 | 1 |
| rs753955 | G |  | 13q12.12 | C/T | 1 | 1 | 1 | 1 |
| rs755383 | T | DMRT1 | 9p24.3 | C/T | 1 | 0 | 1 | 0 |
| rs7554607 | A | RYR2 | 1q43 | A/G | 1 | 1 | 1 | 1 |
| rs757210 | G | HNF1B | 17q12 | A/G | 1 | 0 | 1 | 0 |
| rs7574865 | G | STAT4 | 2q32.3 | G/T | 1 | 1 | 1 | 1 |
| rs7578361 | C |  | 2q23.2 | G/T | 0 | 0 | 0 | 0 |
| rs757978 | T | FARP2 | 2q37.3 | A/G | 1 | 1 | 1 | 1 |
| rs7584330 | C | MLPH | 2q37.3 | A/G | 1 | 0 | 1 | 0 |
| rs7591996 | C |  | 2p25.2 | A/C | 1 | 1 | 1 | 1 |
| rs7611694 | A | SIDT1 | 3q13.2 | A/C | 1 | 1 | 1 | 1 |
| rs7626795 | G | IL1RAP | 3q28 | A/G | 1 | 1 | 1 | 1 |
| rs7629490 | T |  | 3p11.2 | C/T | 1 | 1 | 1 | 1 |
| rs7716600 | A |  | 5p12 | A/C | 1 | 1 | 1 | 1 |
| rs7745098 | G |  | 6q23.3 | C/T | 1 | 1 | 1 | 1 |
| rs7758229 | T | SLC22A3 | 6q25.3 | G/T | 1 | 1 | 1 | 1 |
| rs7832232 | A |  | 8p11.22 | A/G | 1 | 1 | 1 | 1 |
| rs78378222 | C | TP53 | 17p13.1 | A/C | 1 | 1 | 1 | 1 |
| rs7853844 | A | 1-Dec | 9q33.1 | A/G | 1 | 1 | 1 | 1 |
| rs790356 | G | DLG2 | 11q14.1 | A/G | 1 | 1 | 1 | 1 |
| rs7904519 | G | TCF7L2 | 10q25.2 | A/G | 1 | 0 | 1 | 0 |
| rs7931342 | G |  | 11q13.3 | G/T | 1 | 0 | 1 | 0 |
| rs7944004 | T | C11orf21 | 11p15.5 | G/T | 1 | 0 | 0 | 0 |
| rs798766 | T | TACC3 | 4p16.3 | C/T | 1 | 1 | 1 | 1 |
| rs8008270 | G | FERMT2 | 14q22.1 | C/T | 1 | 1 | 1 | 1 |
| rs801114 | G |  | 1q42.13 | G/T | 1 | 1 | 1 | 1 |
| rs8015138 | C |  | 14q22.1 | A/C | 1 | 1 | 1 | 1 |
| rs8023845 | G |  | 15q15.1 | G/T | 1 | 0 | 1 | 0 |
| rs8030672 | A |  | 15q23 | A/T | 0 | 0 | 0 | 0 |
| rs8034191 | G | AGPHD1 | 15q25.1 | C/T | 1 | 1 | 1 | 1 |
| rs8048207 | T | CDH8 | 16q21 | C/T | 1 | 1 | 1 | 1 |
| rs8067378 | G | GSDMB | 17q12 | A/G | 1 | 1 | 1 | 1 |
| rs807624 | A |  | 2p24.3 | A/C | 1 | 0 | 1 | 0 |
| rs8100241 | G | ANKLE1 | 19p13.11 | A/G | 1 | 1 | 1 | 1 |
| rs8102137 | C | CCNE1 | 19q12 | C/T | 1 | 1 | 1 | 1 |
| rs8102476 | C | PPP1R14A | 19q13.2 | C/T | 1 | 1 | 1 | 1 |
| rs817826 | C |  | 9q31.2 | C/T | 1 | 1 | 1 | 1 |
| rs865686 | T |  | 9q31.2 | G/T | 1 | 1 | 1 | 1 |
| rs872071 | G | IRF4 | 6p25.3 | A/G | 1 | 1 | 1 | 1 |
| rs889312 | C |  | 5q11.2 | A/C | 1 | 0 | 1 | 0 |
| rs898518 | A | LEF1 | 4q25 | A/C | 1 | 1 | 1 | 1 |
| rs902774 | A |  | 12q13.13 | A/G | 1 | 1 | 1 | 1 |
| rs907611 | A | LSP1 | 11p15.5 | A/G | 1 | 1 | 0 | 0 |
| rs909116 | T | TNNT3 | 11p15.5 | C/T | 1 | 1 | 1 | 1 |
| rs910873 | T | PIGU | 20q11.22 | A/G | 1 | 1 | 1 | 1 |
| rs926070 | A |  |  |  | 1 | 1 | 0 | 0 |
| rs9268853 | C |  |  |  | 1 | 1 | 0 | 0 |
| rs9272105 | A |  |  |  | 1 | 1 | 0 | 0 |
| rs9272535 | A | HLA-DQA1 | 6p21.32 | A/G | 0 | 0 | 0 | 0 |
| rs9273363 | A |  |  |  | 1 | 1 | 0 | 0 |
| rs9275319 | A |  |  |  | 1 | 1 | 0 | 0 |
| rs9275572 | A |  |  |  | 1 | 1 | 0 | 0 |
| rs9290663 | T | KCNMB2 | 3q26.32 | A/T | 0 | 0 | 0 | 0 |
| rs9303196 | T |  | 17p13.2 | A/T | 0 | 0 | 0 | 0 |
| rs9303542 | G | SKAP1 | 17q21.32 | A/G | 1 | 1 | 1 | 1 |
| rs9363918 | A |  | 6q12 | G/T | 1 | 1 | 1 | 1 |
| rs9364554 | T | SLC22A3 | 6q25.3 | C/T | 1 | 0 | 1 | 0 |
| rs9378805 | C | IRF4 | 6p25.3 | A/C | 1 | 0 | 1 | 0 |
| rs9383938 | T |  | 6q25.1 | G/T | 1 | 0 | 1 | 0 |
| rs9383951 | G | ESR1 | 6q25.1 | C/G | 0 | 0 | 0 | 0 |
| rs9387478 | C |  | 6q22.1 | A/C | 1 | 1 | 1 | 1 |
| rs9390123 | A | PHACTR2 | 6q24.2 | C/T | 1 | 1 | 1 | 1 |
| rs941764 | G | CCDC88C | 14q32.11 | A/G | 1 | 1 | 1 | 1 |
| rs9430161 | G | C1orf127 | 1p36.22 | G/T | 1 | 1 | 1 | 1 |
| rs944289 | T |  | 14q13.3 | C/T | 1 | 1 | 1 | 1 |
| rs9485372 | G |  | 6q25.1 | A/G | 1 | 1 | 1 | 1 |
| rs948562 | G | ZFP91 | 11q12.1 | C/T | 1 | 1 | 1 | 1 |
| rs9502893 | G |  | 6p25.3 | C/T | 1 | 1 | 1 | 1 |
| rs9543325 | C |  | 13q22.1 | C/T | 0 | 0 | 0 | 0 |
| rs9557635 | A | NALCN | 13q33.1 | A/G | 1 | 1 | 1 | 1 |
| rs9573163 | G |  | 13q22.1 | C/G | 0 | 0 | 0 | 0 |
| rs9600079 | T |  | 13q22.1 | G/T | 1 | 1 | 1 | 1 |
| rs961253 | A |  | 20p12.3 | A/C | 1 | 1 | 1 | 1 |
| rs9623117 | C | TNRC6B | 22q13.1 | C/T | 1 | 1 | 1 | 1 |
| rs9642880 | T |  | 8q24.21 | G/T | 1 | 1 | 1 | 1 |
| rs965513 | A |  | 9q22.33 | A/G | 1 | 1 | 1 | 1 |
| rs966423 | C | DIRC3 | 2q35 | C/T | 1 | 1 | 1 | 1 |
| rs9693444 | A |  | 8p12 | A/C | 1 | 1 | 1 | 1 |
| rs971074 | G | ADH7 | 4q23 | A/G | 1 | 1 | 1 | 1 |
| rs975334 | C | CNTN4 | 3p26.2 | C/T | 1 | 1 | 1 | 1 |
| rs9783347 | A | GTF2H1 | 11p15.1 | A/G | 1 | 1 | 1 | 1 |
| rs9790517 | T | TET2 | 4q24 | C/T | 1 | 1 | 1 | 1 |
| rs981782 | C | HCN1 | 5p12 | G/T | 1 | 1 | 1 | 1 |
| rs9868873 | G | SEMA5B | 3q21.1 | A/G | 1 | 1 | 1 | 1 |
| rs9874556 | A |  | 3p26.2 | A/G | 1 | 1 | 1 | 1 |
| rs9929218 | G | CDH1 | 16q22.1 | A/G | 1 | 1 | 1 | 1 |
| rs9934948 | C |  | 16q22.3 | C/T | 1 | 1 | 1 | 1 |
| rs9958208 | A | RIT2 | 18q12.3 | A/G | 1 | 1 | 1 | 1 |
| rs9981861 | G | DSCAM | 21q22.2 | C/T | 1 | 1 | 1 | 1 |
| rs999737 | C | RAD51L1 | 14q24.1 | C/T | 1 | 1 | 1 | 1 |

| **Table S5:** Characteristics of New England Centenarian Study Controls (N=281) | | |
| --- | --- | --- |
| Alive/Deceased^1^ | Mean Age^2^ | Male/Female |
| 189/70 | 78.3 | 161/120 |

1 – 22 Controls were lost to follow-up
 2 – Mean age at death or date of last contact

| **Table S6**: Results of Poisson GLM Ignoring Family Correlation – LLFS Data | | | | | | | | | | | | | |
| --- | --- | --- | --- | --- | --- | --- | --- | --- | --- | --- | --- | --- | --- |
| Alzheimer’s Disease | | | | | | | | | | | | | |
|  | LD threshold of r^2^ > 0.8  (93 SNPs) | | | | | LD threshold of r^2^ > 0.2  (83 SNPs) | | | | Published GRS  (8 SNPs) | | | |
| Data | β^1^ | | Z-Stat | | pval | β^1^ | Z-stat | | pval | β^1^ | Z-Stat | | pval |
| Generation One | -0.01520 | | -1.53 | | 0.125 | -0.00622 | -0.61 | | 0.545 | -0.02261 | -0.73 | | 0.465 |
| Generation Two | -0.00975 | | -1.91 | | 0.056 | -0.00471 | -0.89 | | 0.373 | -0.01231 | -0.77 | | 0.442 |
| Both Generations | -0.01114 | | -2.47 | | 0.013 | -0.00529 | -1.13 | | 0.258 | -0.01635 | -1.16 | | 0.248 |
| CVD and Stroke | | | | | | | | | | | | | |
|  | LD threshold of r^2^ > 0.8  (239 SNPs) | | | | | LD threshold of r^2^ > 0.2  (218 SNPs) | | | | Published GRS  (20 SNPs) | | | |
| Data | β^1^ | | Z-Stat | | pval | β^1^ | Z-stat | | pval | β^1^ | Z-Stat | | pval |
| Generation One | -0.00361 | | -0.62 | | 0.534 | -0.00380 | -0.62 | | 0.533 | 0.02636 | 1.36 | | 0.173 |
| Generation Two | -0.00022 | | -0.07 | | 0.942 | -0.00134 | -0.43 | | 0.669 | 0.00527 | 0.53 | | 0.595 |
| Both Generations | -0.00105 | | -0.40 | | 0.693 | -0.00203 | -0.73 | | 0.463 | 0.00899 | 1.03 | | 0.305 |
| Type 2 Diabetes | | | | | | | | | | | | | |
|  | LD threshold of r^2^ > 0.8  (155 SNPs) | | | | | LD threshold of r^2^ > 0.2  (137 SNPs) | | | | Published GRS  (14 SNPs) | | | |
| Data | β^1^ | | Z-Stat | | pval | β^1^ | Z-stat | | pval | β^1^ | Z-Stat | | pval |
| Generation One | 0.00319 | | 0.48 | | 0.629 | 0.00376 | 0.53 | | 0.595 | 0.01409 | 0.64 | | 0.523 |
| Generation Two | 0.00486 | | 1.43 | | 0.153 | 0.00401 | 1.10 | | 0.270 | 0.01397 | 1.23 | | 0.219 |
| Both Generations | 0.00459 | | 1.53 | | 0.127 | 0.00406 | 1.26 | | 0.206 | 0.01512 | 1.51 | | 0.132 |
| Cancer | | | | | | | | | | | | | |
|  | | LD threshold of r^2^ > 0.8  (431 SNPs) | | | | | | LD threshold of r^2^ > 0.2  (386 SNPs) | | | | | |
| Data | | β^1^ | | Z-Stat | | pval | | β^1^ | | Z-stat | | pval | |
| Generation One | | -0.00074 | | -0.17 | | 0.862 | | -0.00161 | | -0.36 | | 0.718 | |
| Generation Two | | 0.00040 | | 0.18 | | 0.855 | | -0.00052 | | -0.23 | | 0.820 | |
| Both Generations | | 0.00039 | | 0.20 | | 0.842 | | -0.00051 | | -0.25 | | 0.803 | |

1 – β Estimate is for the regression coefficient for the familial longevity indicator (0=control, 1=proband or relative of proband), in log-scale for the analysis of generation one subjects (N=1562), generation two subjects (N=3102), and aggregated data from both generations. The results of generation one and two are adjusted for sex. The results of aggregated data from both generations are adjusted for sex and generation.

| **Table S7**: Results of Poisson GLM with GEE using Exchangeable Correlation Structure – LLFS Data | | | | | | | | | | | | | |
| --- | --- | --- | --- | --- | --- | --- | --- | --- | --- | --- | --- | --- | --- |
| Alzheimer’s Disease | | | | | | | | | | | | | |
|  | LD threshold of r^2^ > 0.8  (93 SNPs) | | | | | LD threshold of r^2^ > 0.2  (83 SNPs) | | | | Published GRS  (8 SNPs) | | | |
| Data | β^1^ | | Z-Stat | | pval | β^1^ | Z-stat | | pval | β^1^ | Z-Stat | | pval |
| Generation One | -0.01668 | | -2.18 | | 0.029 | -0.00801 | -1.13 | | 0.258 | -0.02190 | -0.91 | | 0.365 |
| Generation Two | -0.00929 | | -2.11 | | 0.034 | -0.00390 | -0.98 | | 0.327 | -0.01097 | -0.78 | | 0.435 |
| Both Generations | -0.01037 | | -2.54 | | 0.011 | -0.00433 | -1.16 | | 0.245 | -0.01568 | -1.23 | | 0.219 |
| CVD and Stroke | | | | | | | | | | | | | |
|  | LD threshold of r^2^ > 0.8  (239 SNPs) | | | | | LD threshold of r^2^ > 0.2  (218 SNPs) | | | | Published GRS  (20 SNPs) | | | |
| Data | β^1^ | | Z-Stat | | pval | β^1^ | Z-stat | | pval | β^1^ | Z-Stat | | pval |
| Generation One | -0.00407 | | -1.02 | | 0.305 | -0.00456 | -1.12 | | 0.263 | 0.02737 | 2.05 | | 0.041 |
| Generation Two | 0.00043 | | 0.18 | | 0.855 | -0.00076 | -0.32 | | 0.750 | 0.00475 | 0.63 | | 0.529 |
| Both Generations | -0.00067 | | -0.31 | | 0.756 | -0.00175 | -0.80 | | 0.423 | 0.01118 | 1.59 | | 0.111 |
| Type 2 Diabetes | | | | | | | | | | | | | |
|  | LD threshold of r^2^ > 0.8  (155 SNPs) | | | | | LD threshold of r^2^ > 0.2  (137 SNPs) | | | | Published GRS  (14 SNPs) | | | |
| Data | β^1^ | | Z-Stat | | pval | β^1^ | Z-stat | | pval | β^1^ | Z-Stat | | pval |
| Generation One | 0.00493 | | 1.01 | | 0.310 | 0.00603 | 1.26 | | 0.207 | 0.01594 | 1.08 | | 0.282 |
| Generation Two | 0.00627 | | 2.27 | | 0.023 | 0.00538 | 1.98 | | 0.047 | 0.01707 | 2.12 | | 0.034 |
| Both Generations | 0.00555 | | 2.18 | | 0.029 | 0.00503 | 2.00 | | 0.045 | 0.01745 | 2.32 | | 0.021 |
| Cancer | | | | | | | | | | | | | |
|  | | LD threshold of r^2^ > 0.8  (431 SNPs) | | | | | | LD threshold of r^2^ > 0.2  (386 SNPs) | | | | | |
| Data | | β^1^ | | Z-Stat | | pval | | β^1^ | | Z-stat | | pval | |
| Generation One | | 0.00001 | | 0.003 | | 0.998 | | -0.00090 | | -0.26 | | 0.791 | |
| Generation Two | | 0.00003 | | 0.02 | | 0.985 | | -0.00067 | | -0.39 | | 0.697 | |
| Both Generations | | 0.00015 | | 0.09 | | 0.928 | | -0.00058 | | -0.35 | | 0.729 | |

1 – β Estimate is for the regression coefficient for the familial longevity indicator (0=control, 1=proband or relative of proband), in log-scale for the analysis of generation one subjects (N=1562), generation two subjects (N=3102), and aggregated data from both generations. The results of generation one and two are adjusted for sex. The results of aggregated data from both generations are adjusted for sex and generation.

| **Table S8**: Results of Linear Mixed Model with Random Intercept and Kinship Matrix – LLFS Data | | | | | | | | | | | | | |
| --- | --- | --- | --- | --- | --- | --- | --- | --- | --- | --- | --- | --- | --- |
| Alzheimer’s Disease | | | | | | | | | | | | | |
|  | LD threshold of r^2^ > 0.8  (93 SNPs) | | | | | LD threshold of r^2^ > 0.2  (83 SNPs) | | | | Published GRS  (8 SNPs) | | | |
| Data | β^1^ | | Z-Stat | | pval | β^1^ | Z-stat | | pval | β^1^ | Z-Stat | | pval |
| Generation One | -0.00618 | | -2.20 | | 0.028 | -0.00298 | -1.10 | | 0.271 | -0.01063 | -1.02 | | 0.307 |
| Generation Two | -0.00330 | | -2.13 | | 0.033 | -0.00134 | -0.91 | | 0.363 | -0.00359 | -0.64 | | 0.522 |
| Both Generations | -0.00430 | | -3.30 | | 0.001 | -0.00232 | -1.86 | | 0.063 | -0.00567 | -1.18 | | 0.238 |
| CVD and Stroke | | | | | | | | | | | | | |
|  | LD threshold of r^2^ > 0.8  (239 SNPs) | | | | | LD threshold of r^2^ > 0.2  (218 SNPs) | | | | Published GRS  (20 SNPs) | | | |
| Data | β^1^ | | Z-Stat | | pval | β^1^ | Z-stat | | pval | β^1^ | Z-Stat | | pval |
| Generation One | -0.00136 | | -0.86 | | 0.390 | -0.00158 | -0.98 | | 0.327 | 0.01171 | 2.04 | | 0.041 |
| Generation Two | 0.00041 | | 0.47 | | 0.638 | -0.00024 | -0.27 | | 0.787 | 0.00380 | 1.21 | | 0.226 |
| Both Generations | -0.00020 | | -0.27 | | 0.787 | -0.00075 | -1.00 | | 0.317 | 0.00452 | 1.71 | | 0.088 |
| Type 2 Diabetes | | | | | | | | | | | | | |
|  | LD threshold of r^2^ > 0.8  (155 SNPs) | | | | | LD threshold of r^2^ > 0.2  (137 SNPs) | | | | Published GRS  (14 SNPs) | | | |
| Data | β^1^ | | Z-Stat | | pval | β^1^ | Z-stat | | pval | β^1^ | Z-Stat | | pval |
| Generation One | 0.00238 | | 1.11 | | 0.267 | 0.00263 | 1.26 | | 0.208 | 0.00833 | 1.21 | | 0.225 |
| Generation Two | 0.00369 | | 3.14 | | 0.002 | 0.00322 | 2.81 | | 0.005 | 0.00863 | 2.32 | | 0.020 |
| Both Generations | 0.00307 | | 3.09 | | 0.002 | 0.00284 | 2.93 | | 0.003 | 0.00845 | 2.68 | | 0.007 |
| Cancer | | | | | | | | | | | | | |
|  | | LD threshold of r^2^ > 0.8  (431 SNPs) | | | | | | LD threshold of r^2^ > 0.2  (386 SNPs) | | | | | |
| Data | | β^1^ | | Z-Stat | | pval | | β^1^ | | Z-stat | | pval | |
| Generation One | | 0.00007 | | 0.05 | | 0.959 | | -0.00029 | | -0.22 | | 0.826 | |
| Generation Two | | 0.00076 | | 1.05 | | 0.294 | | 0.00024 | | 0.34 | | 0.732 | |
| Both Generations | | 0.00095 | | 1.56 | | 0.119 | | 0.00058 | | 0.96 | | 0.339 | |

1 – β Estimate is for the regression coefficient for the familial longevity indicator (0=control, 1=proband or relative of proband), in linear scale for the analysis of generation one subjects (N=1562), generation two subjects (N=3102), and aggregated data from both generations. The results of generation one and two are adjusted for sex. The results of aggregated data from both generations are adjusted for sex and generation.

| **Table S9**: Results of Poisson Generalized Linear Mixed Model with Random Intercept per Family – LLFS & NECS Data | | | | | | | | | | | | | |
| --- | --- | --- | --- | --- | --- | --- | --- | --- | --- | --- | --- | --- | --- |
| Alzheimer’s Disease | | | | | | | | | | | | | |
|  | LD threshold of r^2^ > 0.8  (81 SNPs) | | | | | LD threshold of r^2^ > 0.2  (73 SNPs) | | | | Published GRS  (8 SNPs) | | | |
| Data | β^1^ | | Z-Stat | | pval | β^1^ | Z-stat | | pval | β^1^ | Z-Stat | | pval |
| Generation One | -0.01201 | | -1.41 | | 0.159 | -0.00185 | -0.21 | | 0.834 | -0.00303 | -0.12 | | 0.905 |
| Generation Two | -0.00946 | | -1.78 | | 0.076 | -0.00609 | -1.11 | | 0.267 | -0.01230 | -0.76 | | 0.447 |
| Both Generations | -0.01047 | | -2.29 | | 0.022 | -0.00558 | -1.19 | | 0.234 | -0.00956 | -0.69 | | 0.490 |
| CVD and Stroke | | | | | | | | | | | | | |
|  | LD threshold of r^2^ > 0.8  (205 SNPs) | | | | | LD threshold of r^2^ > 0.2  (187 SNPs) | | | | Published GRS  (17 SNPs) | | | |
| Data | β^1^ | | Z-Stat | | pval | β^1^ | Z-stat | | pval | β^1^ | Z-Stat | | pval |
| Generation One | 0.00110 | | 0.21 | | 0.831 | 0.00190 | 0.351 | | 0.726 | 0.02291 | 1.28 | | 0.201 |
| Generation Two | -0.00090 | | -0.27 | | 0.783 | -0.00200 | -0.58 | | 0.556 | 0.00307 | 0.27 | | 0.787 |
| Both Generations | -0.00054 | | -0.19 | | 0.847 | -0.00110 | -0.38 | | 0.705 | 0.00700 | 0.72 | | 0.471 |
| Type 2 Diabetes | | | | | | | | | | | | | |
|  | LD threshold of r^2^ > 0.8  (140 SNPs) | | | | | LD threshold of r^2^ > 0.2  (123 SNPs) | | | | Published GRS  (14 SNPs) | | | |
| Data | β^1^ | | Z-Stat | | pval | β^1^ | Z-stat | | pval | β^1^ | Z-Stat | | pval |
| Generation One | 0.00102 | | 0.18 | | 0.858 | 0.00198 | 0.32 | | 0.746 | 0.02868 | 1.59 | | 0.112 |
| Generation Two | 0.00578 | | 1.58 | | 0.114 | 0.00524 | 1.34 | | 0.180 | 0.01397 | 1.23 | | 0.219 |
| Both Generations | 0.00425 | | 1.36 | | 0.173 | 0.00413 | 1.24 | | 0.214 | 0.01828 | 1.90 | | 0.058 |
| Cancer | | | | | | | | | | | | | |
|  | | LD threshold of r^2^ > 0.8  (377 SNPs) | | | | | | LD threshold of r^2^ > 0.2  (336 SNPs) | | | | | |
| Data | | β^1^ | | Z-Stat | | pval | | β^1^ | | Z-stat | | pval | |
| Generation One | | 0.00355 | | 0.97 | | 0.331 | | 0.00374 | | 0.97 | | 0.330 | |
| Generation Two | | 0.00066 | | 0.28 | | 0.777 | | 0.00021 | | 0.09 | | 0.932 | |
| Both Generations | | 0.00130 | | 0.65 | | 0.514 | | 0.00104 | | 0.50 | | 0.616 | |

1 - β Estimate is for the regression coefficient for the familial longevity indicator (0=control, 1=proband or relative of proband), in log-scale for the analysis of generation one subjects (LLFS N=1387, NECS N=281), generation two subjects (N=3102), and aggregated data from both generations. The results of generation one and two are adjusted for sex. The results of aggregated data from both generations are adjusted for sex and generation.

| **Table S10**: Results of Poisson GLM Ignoring Family Correlation – LLFS & NECS Data | | | | | | | | | | | | | |
| --- | --- | --- | --- | --- | --- | --- | --- | --- | --- | --- | --- | --- | --- |
| Alzheimer’s Disease | | | | | | | | | | | | | |
|  | LD threshold of r^2^ > 0.8  (81 SNPs) | | | | | LD threshold of r^2^ > 0.2  (73 SNPs) | | | | Published GRS  (8 SNPs) | | | |
| Data | β^1^ | | Z-Stat | | pval | β^1^ | Z-stat | | pval | β^1^ | Z-Stat | | pval |
| Generation One | -0.01196 | | -1.43 | | 0.153 | -0.00185 | -0.21 | | 0.831 | -0.00297 | -0.12 | | 0.907 |
| Generation Two | -0.00911 | | -1.72 | | 0.085 | -0.00578 | -1.06 | | 0.291 | -0.01231 | -0.77 | | 0.442 |
| Both Generations | -0.00994 | | -2.23 | | 0.026 | -0.00468 | -1.01 | | 0.311 | -0.00981 | -0.73 | | 0.468 |
| CVD and Stroke | | | | | | | | | | | | | |
|  | LD threshold of r^2^ > 0.8  (205 SNPs) | | | | | LD threshold of r^2^ > 0.2  (187 SNPs) | | | | Published GRS  (17 SNPs) | | | |
| Data | β^1^ | | Z-Stat | | pval | β^1^ | Z-stat | | pval | β^1^ | Z-Stat | | pval |
| Generation One | 0.00110 | | 0.21 | | 0.831 | 0.00190 | 0.35 | | 0.726 | 0.02291 | 1.28 | | 0.201 |
| Generation Two | -0.00089 | | -0.28 | | 0.784 | -0.00200 | -0.59 | | 0.559 | 0.00377 | 0.33 | | 0.739 |
| Both Generations | -0.00035 | | -0.13 | | 0.898 | -0.00093 | -0.32 | | 0.748 | 0.00923 | 0.97 | | 0.334 |
| Type 2 Diabetes | | | | | | | | | | | | | |
|  | LD threshold of r^2^ > 0.8  (140 SNPs) | | | | | LD threshold of r^2^ > 0.2  (123 SNPs) | | | | Published GRS  (14 SNPs) | | | |
| Data | β^1^ | | Z-Stat | | pval | β^1^ | Z-stat | | pval | β^1^ | Z-Stat | | pval |
| Generation One | 0.00102 | | 0.18 | | 0.858 | 0.00198 | 0.32 | | 0.746 | 0.02868 | 1.59 | | 0.111 |
| Generation Two | 0.00669 | | 1.85 | | 0.064 | 0.00597 | 1.54 | | 0.124 | 0.01397 | 1.23 | | 0.219 |
| Both Generations | 0.00511 | | 1.67 | | 0.095 | 0.00488 | 1.49 | | 0.136 | 0.01833 | 1.91 | | 0.056 |
| Cancer | | | | | | | | | | | | | |
|  | | LD threshold of r^2^ > 0.8  (377 SNPs) | | | | | | LD threshold of r^2^ > 0.2  (336 SNPs) | | | | | |
| Data | | β^1^ | | Z-Stat | | pval | | β^1^ | | Z-stat | | pval | |
| Generation One | | 0.00355 | | 0.97 | | 0.331 | | 0.00374 | | 0.97 | | 0.330 | |
| Generation Two | | 0.00084 | | 0.36 | | 0.717 | | 0.00021 | | 0.09 | | 0.932 | |
| Both Generations | | 0.00166 | | 0.85 | | 0.396 | | 0.00126 | | 0.61 | | 0.540 | |

1 – β Estimate is for the regression coefficient for the familial longevity indicator (0=control, 1=proband or relative of proband), in log-scale for the analysis of generation one subjects (LLFS N=1387, NECS N=281), generation two subjects (N=3102), and aggregated data from both generations. The results of generation one and two are adjusted for sex. The results of aggregated data from both generations are adjusted for sex and generation.

| **Table S11**: Results of Poisson GLM with GEE using Exchangeable Correlation Structure – LLFS & NECS Data | | | | | | | | | | | | | |
| --- | --- | --- | --- | --- | --- | --- | --- | --- | --- | --- | --- | --- | --- |
| Alzheimer’s Disease | | | | | | | | | | | | | |
|  | LD threshold of r^2^ > 0.8  (81 SNPs) | | | | | LD threshold of r^2^ > 0.2  (73 SNPs) | | | | Published GRS  (8 SNPs) | | | |
| Data | β^1^ | | Z-Stat | | pval | β^1^ | Z-stat | | pval | β^1^ | Z-Stat | | pval |
| Generation One | -0.01251 | | -1.76 | | 0.078 | -0.00293 | -0.45 | | 0.653 | -0.00608 | -0.30 | | 0.764 |
| Generation Two | -0.00973 | | -2.09 | | 0.036 | -0.00729 | -1.67 | | 0.095 | -0.01261 | -0.87 | | 0.386 |
| Both Generations | -0.01062 | | -2.52 | | 0.012 | -0.00667 | -1.67 | | 0.095 | -0.01009 | -0.79 | | 0.429 |
| CVD and Stroke | | | | | | | | | | | | | |
|  | LD threshold of r^2^ > 0.8  (205 SNPs) | | | | | LD threshold of r^2^ > 0.2  (187 SNPs) | | | | Published GRS  (17 SNPs) | | | |
| Data | β^1^ | | Z-Stat | | pval | β^1^ | Z-stat | | pval | β^1^ | Z-Stat | | pval |
| Generation One | 0.00160 | | 0.42 | | 0.672 | 0.00189 | 0.49 | | 0.624 | 0.02176 | 1.57 | | 0.118 |
| Generation Two | -0.00123 | | -0.44 | | 0.663 | -0.00239 | -0.81 | | 0.417 | -0.00095 | -0.10 | | 0.930 |
| Both Generations | -0.00087 | | -0.33 | | 0.740 | -0.00154 | -0.58 | | 0.560 | 0.00323 | 0.35 | | 0.729 |
| Type 2 Diabetes | | | | | | | | | | | | | |
|  | LD threshold of r^2^ > 0.8  (140 SNPs) | | | | | LD threshold of r^2^ > 0.2  (123 SNPs) | | | | Published GRS  (14 SNPs) | | | |
| Data | β^1^ | | Z-Stat | | pval | β^1^ | Z-stat | | pval | β^1^ | Z-Stat | | pval |
| Generation One | 0.00372 | | 0.94 | | 0.348 | 0.00470 | 1.17 | | 0.242 | 0.03054 | 2.51 | | 0.012 |
| Generation Two | 0.00422 | | 1.06 | | 0.290 | 0.00363 | 0.88 | | 0.381 | 0.01355 | 1.48 | | 0.138 |
| Both Generations | 0.00381 | | 1.06 | | 0.290 | 0.00341 | 0.92 | | 0.357 | 0.01771 | 2.16 | | 0.031 |
| Cancer | | | | | | | | | | | | | |
|  | | LD threshold of r^2^ > 0.8  (377 SNPs) | | | | | | LD threshold of r^2^ > 0.2  (336 SNPs) | | | | | |
| Data | | β^1^ | | Z-Stat | | pval | | β^1^ | | Z-stat | | pval | |
| Generation One | | 0.00388 | | 1.35 | | 0.179 | | 0.00392 | | 1.39 | | 0.166 | |
| Generation Two | | -0.00017 | | -0.08 | | 0.934 | | -0.00072 | | -0.37 | | 0.715 | |
| Both Generations | | 0.00079 | | 0.42 | | 0.674 | | 0.00042 | | 0.24 | | 0.812 | |

1 – β Estimate is for the regression coefficient for the familial longevity indicator (0=control, 1=proband or relative of proband), in log-scale for the analysis of generation one subjects (LLFS N=1387, NECS N=281), generation two subjects (N=3102), and aggregated data from both generations. The results of generation one and two are adjusted for sex. The results of aggregated data from both generations are adjusted for sex and generation.

| **Table S12**: Results of Linear Mixed Model with Random Intercept and Kinship Matrix – LLFS & NECS Data | | | | | | | | | | | | | |
| --- | --- | --- | --- | --- | --- | --- | --- | --- | --- | --- | --- | --- | --- |
| Alzheimer’s Disease | | | | | | | | | | | | | |
|  | LD threshold of r^2^ > 0.8  (81 SNPs) | | | | | LD threshold of r^2^ > 0.2  (73 SNPs) | | | | Published GRS  (8 SNPs) | | | |
| Data | β^1^ | | Z-Stat | | pval | β^1^ | Z-stat | | pval | β^1^ | Z-Stat | | pval |
| Generation One | -0.00478 | | -1.81 | | 0.070 | -0.00112 | -0.44 | | 0.660 | -0.00236 | -0.27 | | 0.787 |
| Generation Two | -0.00299 | | -1.79 | | 0.073 | -0.00161 | -0.99 | | 0.322 | -0.00359 | -0.64 | | 0.522 |
| Both Generations | -0.00334 | | -2.25 | | 0.024 | -0.00150 | -1.04 | | 0.300 | -0.00276 | -0.55 | | 0.582 |
| CVD and Stroke | | | | | | | | | | | | | |
|  | LD threshold of r^2^ > 0.8  (205 SNPs) | | | | | LD threshold of r^2^ > 0.2  (187 SNPs) | | | | Published GRS  (17 SNPs) | | | |
| Data | β^1^ | | Z-Stat | | pval | β^1^ | Z-stat | | pval | β^1^ | Z-Stat | | pval |
| Generation One | 0.00068 | | 0.46 | | 0.644 | 0.00080 | 0.53 | | 0.599 | 0.00887 | 1.65 | | 0.098 |
| Generation Two | 0.00020 | | 0.21 | | 0.834 | -0.00045 | -0.46 | | 0.646 | 0.00335 | 0.99 | | 0.323 |
| Both Generations | 0.00025 | | 0.30 | | 0.767 | -0.00021 | -0.25 | | 0.805 | 0.00464 | 1.54 | | 0.123 |
| Type 2 Diabetes | | | | | | | | | | | | | |
|  | LD threshold of r^2^ > 0.8  (140 SNPs) | | | | | LD threshold of r^2^ > 0.2  (123 SNPs) | | | | Published GRS  (14 SNPs) | | | |
| Data | β^1^ | | Z-Stat | | pval | β^1^ | Z-stat | | pval | β^1^ | Z-Stat | | pval |
| Generation One | 0.00166 | | 0.86 | | 0.390 | 0.00212 | 1.11 | | 0.267 | 0.01503 | 2.57 | | 0.010 |
| Generation Two | 0.00457 | | 3.60 | | .0003 | 0.00416 | 3.34 | | 0.001 | 0.00863 | 2.32 | | 0.020 |
| Both Generations | 0.00410 | | 3.69 | | .0002 | 0.00390 | 3.57 | | .0004 | 0.01036 | 3.13 | | 0.002 |
| Cancer | | | | | | | | | | | | | |
|  | | LD threshold of r^2^ > 0.8  (377 SNPs) | | | | | | LD threshold of r^2^ > 0.2  (336 SNPs) | | | | | |
| Data | | β^1^ | | Z-Stat | | pval | | β^1^ | | Z-stat | | pval | |
| Generation One | | 0.00168 | | 1.36 | | 0.174 | | 0.00173 | | 1.40 | | 0.162 | |
| Generation Two | | 0.00099 | | 1.26 | | 0.208 | | 0.00058 | | 0.74 | | 0.459 | |
| Both Generations | | 0.00124 | | 1.78 | | 0.075 | | 0.00096 | | 1.37 | | 0.171 | |

1 – β Estimate is for the regression coefficient for the familial longevity indicator (0=control, 1=proband or relative of proband), in linear scale for the analysis of generation one subjects (LLFS N=1387, NECS N=281), generation two subjects (N=3102), and aggregated data from both generations. The results of generation one and two are adjusted for sex. The results of aggregated data from both generations are adjusted for sex and generation.


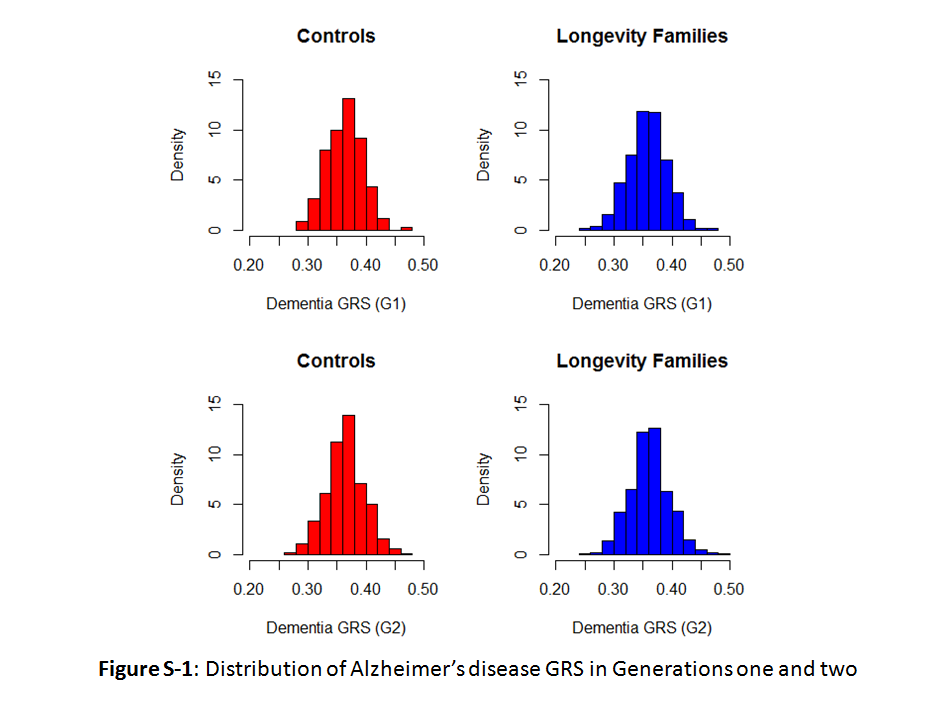


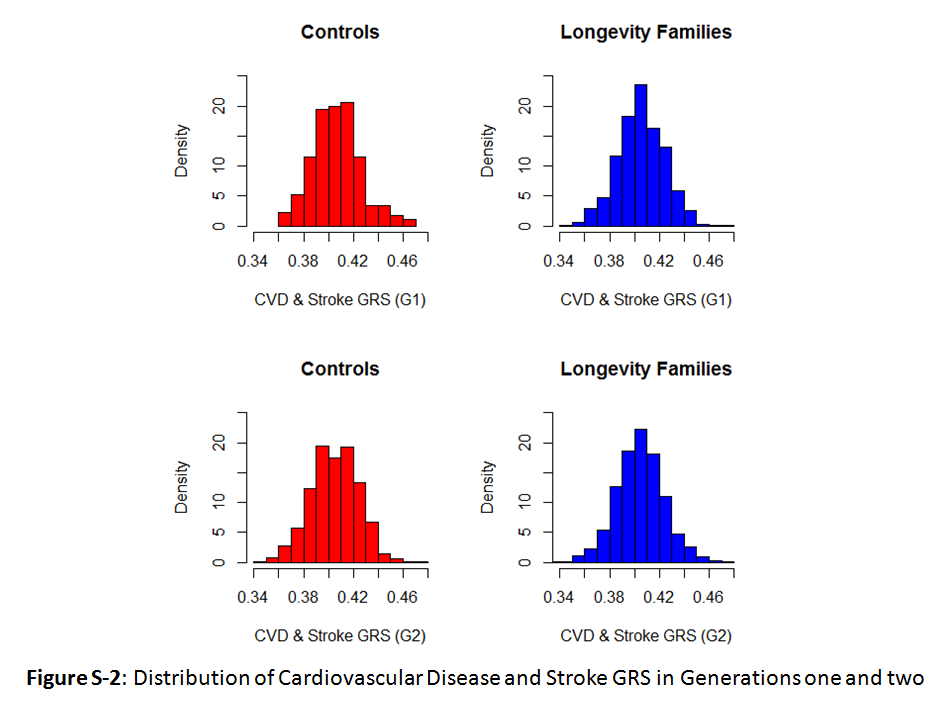


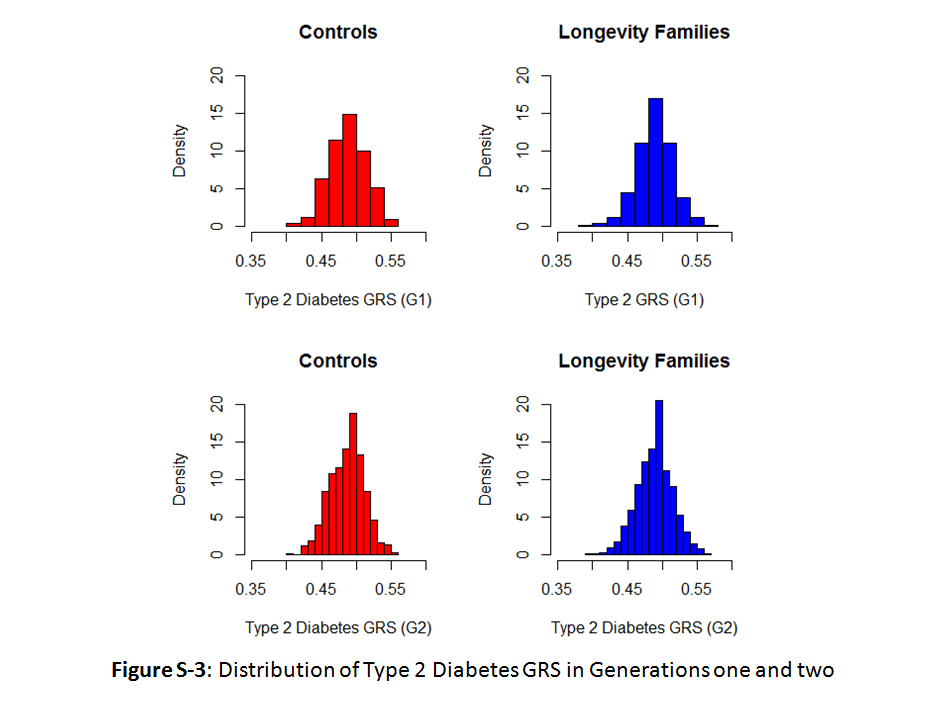


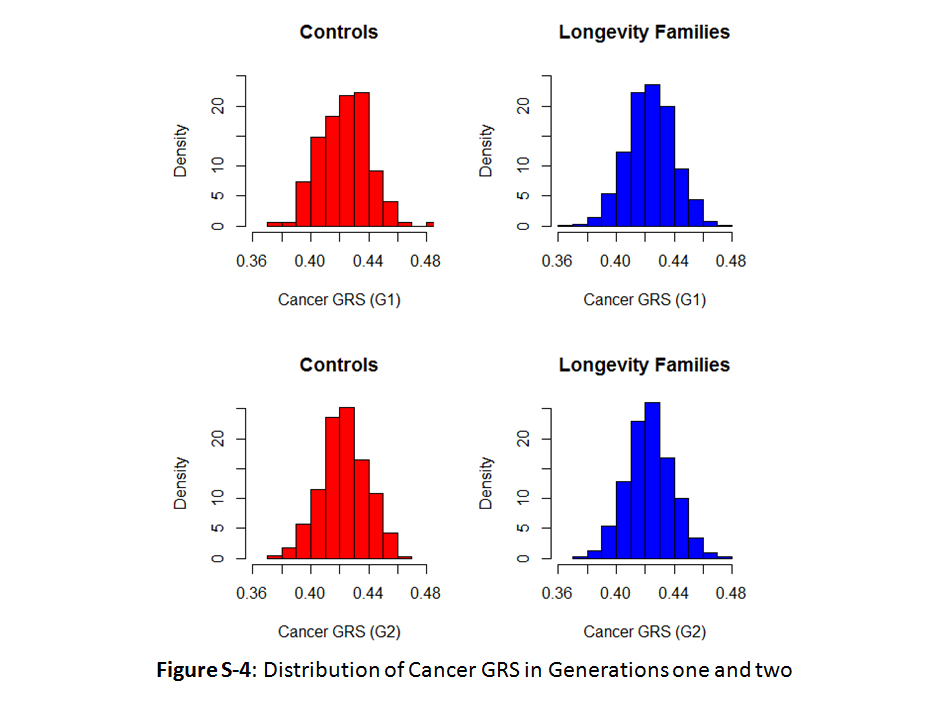

Supplement: Supplementary file 1 [file aging-07-0123-s001.docx]
